# A Network Meta-Analysis of Cancer Immunotherapies *Versus* Chemotherapy for First-Line Treatment of Patients With Non-Small Cell Lung Cancer and High Programmed Death-Ligand 1 Expression

**DOI:** 10.3389/fonc.2021.676732

**Published:** 2021-07-09

**Authors:** Roy Herbst, Jacek Jassem, Seye Abogunrin, Daniel James, Rachael McCool, Rossella Belleli, Giuseppe Giaccone, Filippo De Marinis

**Affiliations:** ^1^ Yale Comprehensive Cancer Center, Yale School of Medicine, New Haven, CT, United States; ^2^ Dept. of Oncology and Radiotherapy, Medical University of Gdańsk, Gdańsk, Poland; ^3^ Access Centre of Excellence, Global Access, F. Hoffmann-La Roche, Basel, Switzerland; ^4^ Quantics Biostatistics, Edinburgh, United Kingdom; ^5^ York Health Economics Consortium Ltd, University of York, York, United Kingdom; ^6^ Sandra and Edward Meyer Cancer Center, Weill-Cornell Medicine, New York, NY, United States; ^7^ Division of Thoracic Oncology, European Institute of Oncology, Milan, Italy

**Keywords:** non-small cell lung cancer, immunotherapy, atezolizumab, pembrolizumab, nivolumab, ipilimumab, meta-analysis

## Abstract

In the absence of head-to-head trials of first-line treatments for metastatic non-small cell lung cancer (NSCLC), synthesis of available evidence is needed. We conducted a systematic literature review and network meta-analysis of randomized controlled trials in patients with stage IV NSCLC and high programmed death-ligand 1 (PD-L1) expression. Patients with other-stage NSCLC or without PD-L1 expression and populations with < 80% stage IV NSCLC were excluded. Outcomes included overall survival (OS), progression-free survival (PFS), objective response rate (ORR), and treatment-related adverse events. English records from MEDLINE and Embase published through October 2020 were eligible, supplemented by hand searches of other sources. Three evidence networks were constructed based on histology (mixed, squamous, non-squamous). OS and PFS results were analyzed applying Bayesian fractional polynomial random-effects models. Hazard ratios over time with 95% credible intervals (CrIs) and expected differences in OS and PFS between each cancer immunotherapy regimen and the chemotherapy common comparator were generated. Seventeen clinical trials were included after screening 32,527 records. Heterogeneity and risk of bias were generally low across trials. In the mixed-histology network of PD-L1–high patients, expected OS was significantly longer with atezolizumab (estimated difference: 10.4 months [95% CrI: 1.9, 18.2]), pembrolizumab (7.2 [2.2, 12.3]), and cemiplimab (13.0 [4.2, 21.0]) *versus* chemotherapy but not with nivolumab (3.5 [−2.5, 10.6]) or nivolumab plus ipilimumab (6.7 [−0.5, 14.2]) *versus* chemotherapy. OS improvements were not significant compared with chemotherapy for any regimen in the squamous and non-squamous networks, except pembrolizumab plus chemotherapy in the non-squamous network. All regimens showed significantly longer expected PFS *versus* chemotherapy in the non-squamous network, whereas the increases were not significant in the mixed or squamous networks. ORR was significantly higher with pembrolizumab and cemiplimab *versus* chemotherapy in the mixed-histology network, with sintilimab in the non-squamous network, and with combination regimens, including pembrolizumab or atezolizumab, in the squamous and non-squamous networks, except with atezolizumab plus carboplatin, paclitaxel, and bevacizumab. Survival and safety *versus* chemotherapy were generally similar across cancer immunotherapies and histology networks. These findings may support treatment decisions for patients with high PD-L1 status receiving first-line treatment for NSCLC.

## Introduction

Non-small cell lung cancer (NSCLC) constitutes approximately 85% of all lung cancers and is the leading cause of cancer-related death (1, 2). The advent of cancer immunotherapy (CIT) and targeted treatments has introduced effective first-line treatment options for metastatic and advanced disease (3, 4). Targeted therapies, such as the anti-vascular endothelial growth factor monoclonal antibody bevacizumab, are recommended in certain combination regimens regardless of programmed death-ligand 1 (PD-L1) status (3). Immunohistochemistry testing is recommended to determine the suitability for first-line CIT based on the level of PD-L1 expression, the patient’s health status, and clinical circumstances (3, 4).

Single-agent and combination first-line CIT has been investigated in several phase III clinical trials, and clinical practice recommendations factor in levels of PD-L1 expression and squamous or non-squamous histology. The anti–programmed death-1 inhibitors pembrolizumab and nivolumab, including nivolumab in combination with the CTLA-4 inhibitor ipilimumab, and the PD-L1 inhibitor atezolizumab are all recommended for first-line use in different clinical scenarios (3–8). CIT regimens may be used as monotherapy or in combination with chemotherapy or other regimens based on treatment history, genetic alterations, histology, and level of PD-L1 expression (3, 4).

The pace of cancer treatment research and the complexity of yet-evolving biomarker testing make clinical and policy decision making challenging—and the process is further complicated by the lack of head-to-head comparisons among standards of care and emerging treatment options. Several attempts have been made to provide meaningful indirect comparisons, but these either have been limited in scope or have not included data from all relevant CIT trials (9–11). Against this background, and to accommodate CIT-specific considerations with appropriate statistical methodology and the most recent clinical trial findings, we conducted a network meta-analysis (NMA) of CIT monotherapy and combination regimens for patients with metastatic NSCLC.

## Methods

We conducted a systematic literature review (SLR) of randomized controlled trials (phase II, III, or IV) including adults (≥ 18 years) with stage IV squamous or non-squamous NSCLC and no prior chemotherapy for stage IV disease. This analysis focused on patients whose tumors had high PD-L1 expression (defined as a tumor proportion score ≥ 50% or as either ≥ 50% of tumor cells [TC; TC3] or ≥ 10% of tumor-infiltrating immune cells [IC; IC3]). Trials that were not limited to patients with stage IV NSCLC were eligible if > 80% of the population had stage IV disease. Interventions of interest included monotherapy or combination therapy with CIT (atezolizumab, pembrolizumab, nivolumab, ipilimumab, durvalumab), bevacizumab, tremelimumab, or chemotherapy (carboplatin, cisplatin, docetaxel, etoposide, gemcitabine, *nab*-paclitaxel, paclitaxel, pemetrexed, or vinorelbine).

The systematic literature search was carried out in September and October 2020 in a variety of databases, including Embase (including MEDLINE), PubMed, Cochrane Central Register of Controlled Trials (CENTRAL), Cochrane Database of Systematic Reviews (CDSR), and the Health Technology Assessment database (HTA). The search strategy comprised two main concepts: “NSCLC” AND “RCTs*”*. Full search terms are provided in **Table S1**, and the details of the databases searched are noted in **Table S2**. Additional sources included reference lists from relevant publications and congress abstracts (2012–2020) from the American Society of Clinical Oncology, the European Society for Medical Oncology, the International Association for the Study of Lung Cancer, the World Conference on Lung Cancer, the European Lung Cancer Congress, and the British Thoracic Oncology Group. Clinical trial registries, including ClinicalTrials.gov, the International Clinical Trials Registry Platform, and the European Union Clinical Trials Registry, were searched. We also searched the following health technology assessment websites: the European Medicines Agency, the National Institute for Health and Care Excellence, the Canadian Agency for Drugs and Technologies in Health (including the pan-Canadian Oncology Drug Review), the Independent Institute for Quality and Efficiency in Health Care (IQWiG), and the United States Food and Drug Administration (accesstodata.fda.gov). Two independent reviewers screened titles and abstracts of retrieved records and then the full texts of potentially eligible records, with discrepancies adjudicated by a third reviewer. Detailed data extraction and risk of bias assessment were carried out by two independent reviewers, with discrepancies adjudicated by a third reviewer (**Table S3**).

Included trials were evaluated for the reporting of allocation sequence and concealment, blinding, handling of incomplete outcomes data, selective reporting, and other potential sources of bias. A feasibility assessment was conducted to evaluate the comparability of the patient populations, the outcome measure definitions, and the timing of outcome measures across trials. Heterogeneity was assessed for all treatment comparisons that included two or more studies by visual inspection of the Kaplan-Meier curves, forest plots, and I^2^ statistic for summary measures.

### Systematic Literature Review Results

**Figure 1:**
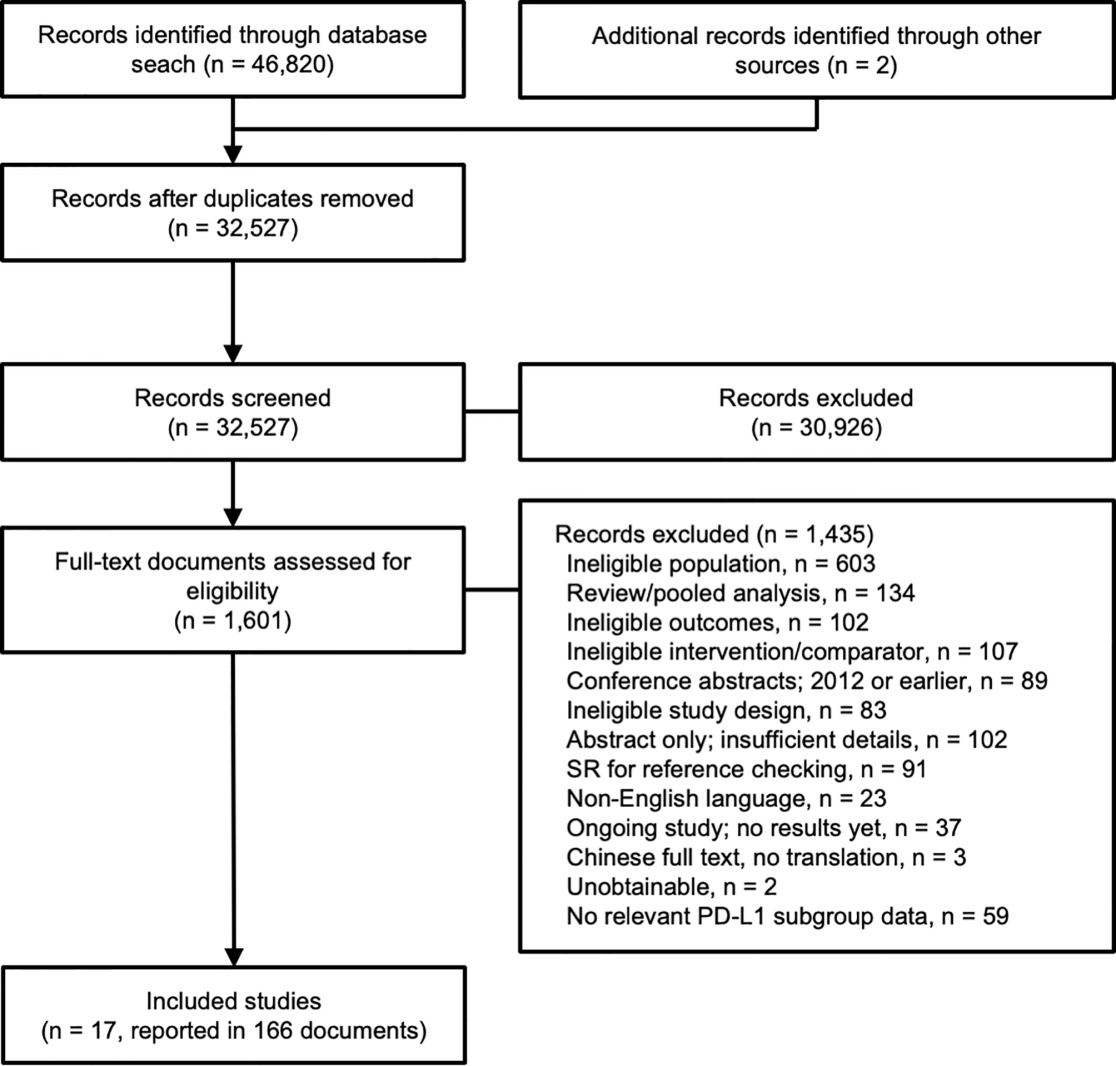
Study flow diagram.

A total of 46,820 records were identified through the searches, and an additional two records were identified from other sources (supplied by the study sponsor, F. Hoffmann-La Roche Ltd). Following de-duplication, 32,527 records were screened according to their titles and abstracts, and 1,601 (5%) records underwent full-text review (**Figure 1**). Seventeen clinical trials reported in 166 publications were included in the evidence networks.

All trials except for KEYNOTE-189, KEYNOTE-407, and ORIENT-11 were open label. None of the trials were designed as crossover trials. However, in nine trials, patients were permitted to receive subsequent CIT or other treatments beyond the trial interventions following disease progression or according to other criteria: CHECKMATE-026—58% of patients in the chemotherapy group received nivolumab at disease progression by the investigator and confirmed by an independent radiologist (12); EMPOWER-LUNG 1—74% of patients who progressed on chemotherapy received subsequent cemiplimab (13); IMpower130—41% of patients in the carboplatin plus nab-paclitaxel group received atezolizumab as monotherapy upon disease progression as assessed by the investigator; a protocol amendment in June 2016 removed this option; KEYNOTE-021, KEYNOTE-024, KEYNOTE-189, and KEYNOTE-407—32%, 44%, 33%, and 27% of patients in these trials, respectively, received pembrolizumab after radiological disease progression or disease progression verified by blinded independent radiological review (5, 14–20); ORIENT-11—27% of patients in the placebo plus chemotherapy group received sintilimab monotherapy during the study after confirmed disease progression, and 5% had received immunotherapy outside the study, which resulted in an effective treatment change rate of 31% (21); and RATIONALE 304—patients in the comparator arm were eligible to receive the intervention after disease progression (22).

### Evidence Networks

Three histology-based evidence networks were constructed: a mixed-histology network, a squamous NSCLC network, and a non-squamous NSCLC network. The mixed-histology network included atezolizumab monotherapy (IMpower110), pembrolizumab monotherapy (KEYNOTE-024, KEYNOTE-042), nivolumab and nivolumab plus ipilimumab (CheckMate-026, CheckMate-227), durvalumab and durvalumab plus tremelimumab (MYSTIC), and cemiplimab (EMPOWER-LUNG 1). We were unable to determine the proportion of patients in the CheckMate-9LA trial with stage IV disease from the available publications; therefore, CheckMate-9LA could not be included in the evidence network. The squamous NSCLC network included atezolizumab monotherapy and combination therapy (IMpower110 subgroup, IMpower131) and pembrolizumab monotherapy and combination therapy (KEYNOTE-024 subgroup, KEYNOTE-042 subgroup, KEYNOTE-407). The non-squamous NSCLC network included atezolizumab monotherapy and combination therapy (IMpower110 subgroup, IMpower130, IMpower132, IMpower150), pembrolizumab monotherapy and combination therapy (KEYNOTE-021, KEYNOTE-024 subgroup, KEYNOTE-042 subgroup, KEYNOTE-189), sintilimab plus chemotherapy (ORIENT-11), and tislelizumab plus chemotherapy (RATIONALE 304).

### Statistical Analysis

Outcomes of interest were overall survival (OS), progression-free survival (PFS), objective response rate (ORR), and safety outcomes, including any treatment-related adverse events (TRAEs). Descriptive statistics were provided for the study and patient characteristics. Individual patient data were available from IMpower110, IMpower130, and IMpower150; summary and subgroup data were available from other included trials. All chemotherapy arms across trials were assumed to be exchangeable (such as the combination of chemotherapy and bevacizumab) and utilized as a single node in the evidence networks (cisplatin and carboplatin were assumed to be equivalent, and studies with and without pemetrexed maintenance therapy were also assumed to be equivalent). Traditional meta-analytic methods require an assumption of proportional hazards, which does not account for the delayed onset or duration of treatment effect observed with CITs (23, 24). Our NMA for time-to-event data (OS and PFS) in the PD-L1–high subgroup used non-proportional hazards fractional polynomial (FP) models within a Bayesian framework (24) using informative priors for the between-study variance of treatment effects (25), allowing the HRs to change over time. Evaluation of the best model for analysis included inspection of predicted survival and observed Kaplan-Meier curves and Deviance Information Criterion measure of model fit. Inspection of the log cumulative hazard plots suggested that the non-proportionality of hazards assumption was upheld. In general, the curves crossed early (maximum, 6 months) compared with the entire observation period. OS and PFS were also analyzed in an NMA using hazard ratios (HRs) assuming proportional hazards to examine the consistency of findings.

We conducted NMAs assuming binomial distribution and logit link for ORR and safety outcomes, in line with the recommendations of the National Institute for Health and Care Excellence (NICE) Decision Support Unit (26). Informative priors were used for the heterogeneity of treatment effects (25). Safety was only analyzed in a mixed-histology network because of the availability of data. Chemotherapy was used as the reference treatment for network comparisons. Results of FP NMAs are presented as HRs over time with 95% credible intervals (CrIs) and expected difference in OS or PFS (60-month time horizons). For the survival outcomes, results from first-order FP random effects models with p1 = 0 (Weibull) are presented based on statistical criteria of goodness-of-fit and clinical plausibility of the survival extrapolations. Results of proportional hazards-based NMAs are presented as HRs and associated 95% CrI. Estimated differences and ratios are considered significant if the 95% Crl lie completely below or above 0 and 1, respectively. All analyses were conducted using R version 3.6.1 in combination with rjags and JAGS version 4.3.0.

## Results

### Study and Patient Characteristics

The characteristics of the 17 studies and inclusive treatments in the analyses for each evidence network are presented in **Table 1**. All included trials had similar eligibility requirements in terms of age, Eastern Cooperative Oncology Group (ECOG) performance status, disease stage, availability of PD-L1 status, and prior chemotherapy for metastatic disease. The analyzed populations of the CheckMate trials, EMPOWER-LUNG 1, IMpower110, IMpower132, KEYNOTE trials, MYSTIC, ORIENT-11, and RATIONALE 304 were restricted to patients with an absence of *EGFR* mutations or *ALK* translocations. IMpower130, IMpower131, and IMpower150 included patients with *EGFR* mutations or *ALK* translocations but required these patients to have progressed on or after appropriate tyrosine kinase inhibitor or *ALK* inhibitor treatment. IMpower130 and IMpower150 reported pre-specified analyses in populations that excluded patients with these mutations; we used the relevant subgroups of patients with PD-L1 ≥ 50% from these populations in our analyses. In the mixed-histology network, the MYSTIC trial only provided a hazard ratio for OS and so could only be included in the proportional hazards HR analysis for that outcome. In the non-squamous network, tislelizumab plus chemotherapy could only be evaluated in the proportional hazards HR analysis of PFS, and sintilimab could be evaluated only in the PFS and ORR analyses.

**Table 1:** Studies and treatments in the evidence network.

Study	Randomized patients	Histology, n (%)	PD-L1 status, n (%)^a^	Network comparator(s)	Data source(s)
IMpower110	554^b^	Squamous: 169 (31) Non-squamous: 385 (69)	TC2/3 or IC2/3: 328 (59) TC3 or IC3: 205 (37) TC1/2/3 or IC1/2/3: 554 (100)	Atezolizumab	**Spigel 2019** (27)**, Herbst 2020** (8) Interim analysis (Sept 2018); median follow-up 17.4 months **Individual patient data**
IMpower150	1202	Non-squamous: 1202 (100)	TC0 and IC0: 584 (49) TC2/3 or IC2/3: 410 (34) TC1/2/3 or IC1/2/3: 617 (51)	Atezolizumab combination	**Roche data on file, May 2018** (28, 29) Median follow-up 20 months for OS and PFS (22 Jan 2018) **Individual patient data**
IMpower130	724	Non-squamous: 724 (100)	TC0 and IC0: 382 (53) TC1/2 or IC1/2: 207 (29) TC3 or IC3: 134 (19) TC1/2/3 or IC1/2/3: 341 (47)	Atezolizumab combination	**Roche data on file, May 2018** (30) Median follow-up 19 months for OS and PFS (15 March 2018) **Individual patient data**
IMpower131	1021	Squamous: 1021 (100)	TC0 and IC0: 501 (49) TC1/2 or IC1/2: 364 (36) TC3 or IC3: 154 (15) TC1/2/3 or IC1/2/3: 518 (51)	Atezolizumab combination	**Jotte 2018** (31)**, Jotte 2020** (32) Median follow-up 17.1 months for PFS (Jan 2018), 26.8 months for OS (Oct 2018) **Digitized summary data**
IMpower132	578	Non-squamous: 578 (100)	TC0 and IC0: 163 (28) TC1/2 or IC1/2: 136 (24) TC3 or IC3: 45 (8) TC1/2/3 or IC1/2/3: 181 (31)	Atezolizumab combination	**Papadimitrakopoulou 2018** (33) Median follow-up 14.8 months (May 2018) **Digitized summary data**
KEYNOTE-021	123	Non-squamous: 123 (100)	< 1%: 44 (36) 1–49%: 42 (34) ≥ 50%: 37 (30)	Pembrolizumab combination	**Langer 2016** (14)**, Borghai 2017** (34) Median follow-up 10.6 months **Digitized summary data**
KEYNOTE-024	305	Squamous: 56 (18) Non-squamous: (82)	≥ 50%: 305 (100)	Pembrolizumab	**Brahmer 2020** (5) Median follow-up 59.9 months (Jun 2020) **Brahmer 2017 (OS, ORR)** (16) Median follow-up 25.2 months (July 2017) for OS and ORR **Reck 2016 (PFS)** (15) Median follow-up 11.2 months (May 2016) **Digitized summary data**
KEYNOTE-042	1274	Squamous: 492 (39) Non-squamous: 782 (61)	≥ 1%: 1274 (100) ≥ 50%: 599 (47)	Pembrolizumab	**Mok 2019a** (6) Second interim analysis (Feb 2018). Median follow-up 12.8 months (IQR 6.0-20.0). KM curve available for OS and PFS **Mok 2019b** (35) Final analysis (Sept 2018); median follow-up 14 months; HR only for OS and PFS for each PD-L1 subgroup **Digitized summary data**
KEYNOTE-189	616	Non-squamous: 616 (100)	< 1%: 190 (31) ≥ 1%: 388 (63) 1–49%: 186 (30) ≥ 50%: 202 (33) Not evaluated: 38 (6)	Pembrolizumab combination	**Gandhi 2018** (17) Median follow-up 10.5 months KM curve available for OS and PFS **Gadgeel 2020** (18) Median follow-up 23.1 months for OS and PFS (Sep 2018) **Digitized summary data**
KEYNOTE-407	559	Squamous: 559 (100)	< 1%: 194 (35) ≥ 1%: 353 (63)	Pembrolizumab combination	**Paz-Ares 2018** (19) Second interim analysis (April 2018) at median follow-up 7.8 months (range 0.1 to 19.1) **Paz-Ares 2020** (20) Median follow-up 14.3 months for OS and ORR **Digitized summary data**
CheckMate-026	541	Squamous: 130 (24) Non-squamous: 411 (76)	≥ 1%: 541 (100) ≥ 50%: 214 (40)	Nivolumab	**Carbone 2017** (12) Final analysis (August 2016) at median follow-up 13.5 months **Digitized summary data**
CheckMate-227	1189	Squamous: 350 (29) Non-squamous: 839 (71)	≥ 1%: 1189 (100) 1–49%: 578 (49) ≥ 50%: 611 (51)	Nivolumab +/- ipilimumab	**Hellmann 2018** (36)**, Hellmann 2019** (37) Minimum follow-up 29.3 months **Digitized summary data**
RATIONALE 304	334	Non-squamous: 334 (100)	< 1%: 144 (43) 1–49%: 80 (24) ≥ 50%: 110 (33)	Tislelizumab combination	**Lu 2020** (22) Interim analysis median follow-up 9.8 months (Jan 2020) **Digitized summary data**
EMPOWER-LUNG 1	710	Squamous: 311 (44) Non-squamous: 399 (56)	≥ 50%: 563 (79)	Cemiplimab monotherapy	**Sezer 2020** (13) Interim analysis; median duration of exposure 27.3 months with cemiplimab and 17.7 months with chemotherapy (Mar 2020) **Digitized summary data**
MYSTIC	1118	Squamous: 320 (29) Non-squamous: 798 (71)	≥ 1%: 864 (77) ≥ 25%: 488 (44) ≥ 50%: 333 (30)	Durvalumab +/- tremelimumab	**Rizvi 2020** (38) Median follow-up 30.2 months for OS (Oct 2018) and 10.6 months for PFS (Jun 2017) **Digitized summary data**
ORIENT-11	397	Non-squamous: 397 (100)	< 1%: 129 (32) ≥ 1%: 268 (68) ≥ 50%: 168 (42)	Sintilimab combination	**Yang 2020** (21) Median follow-up 8.9 months (Nov 2019) **Digitized summary data**

HR, hazard ratio; IQR, interquartile range; KM, Kaplan Meier; OS, overall survival; ORR, objective response rate; PD-L1, programmed death-ligand 1; PFS, progression-free survival.

a PD-L1 status is shown as reported for the study populations. This NMA focused on patients with PD-L1 ≥ 50% or TC3/IC3.

b IMpower110 enrolled 572 patients then excluded 18 patients with an EGFR mutation or ALK translocation; 554 patients with EGFR and ALK wild-type tumors comprised the primary analysis population (ITT-WT) (8, 27).

The median age of patients was similar across trials, ranging from 60 to 66 years. The proportion of males in each treatment arm varied substantially both within and across the trials, ranging from 37% (KEYNOTE-021) to 85% (EMPOWER-LUNG 1). In the trials that reported ethnicity, ≥ 80% of the patients in each treatment arm were White (IMpower150, IMpower130, IMpower131, KEYNOTE-021, CheckMate-026) except in MYSTIC and IMpower132 (66–71% White) and RATIONALE 304 (100% Asian). Six trials did not report the ethnic breakdown of patients (CheckMate-227, IMpower110, KEYNOTE-024, KEYNOTE-042, KEYNOTE-189, KEYNOTE-407). All trials required patients to be chemotherapy naive for metastatic stage IV NSCLC. The proportion of patients with prior adjuvant or neoadjuvant chemotherapy was low: 0% to 11% in the trials reported this information (CheckMate-026, EMPOWER-LUNG 1, KEYNOTE-021, KEYNOTE-024, KEYNOTE-189, KEYNOTE-402, KEYNOTE-407, IMpower130, IMpower150). Approximately 11% to 23% of patients had liver metastases across the trials in which this was reported (CheckMate-026, CheckMate-227, IMpower110, IMpower130, IMpower131, IMpower132, IMpower150, RATIONALE 304).

Similar definitions of OS were used across trials. For PFS and ORR, all trials used RECIST 1.1. Atezolizumab trials and the 60-month follow-up report from KEYNOTE-024 used investigator-assessed PFS and ORR, whereas all other trials used independent review for both outcomes. For the purposes of this analysis, PFS and ORR definitions were assumed to be comparable. Risk of bias was generally low across trials, although all but three were open label (KEYNOTE-189, KEYNOTE-407, ORIENT-11). Blinding of the outcome assessor was unclear for the atezolizumab trials (**Table S4** **).**

### NMA Results: Overall Survival

**Figure 2:**
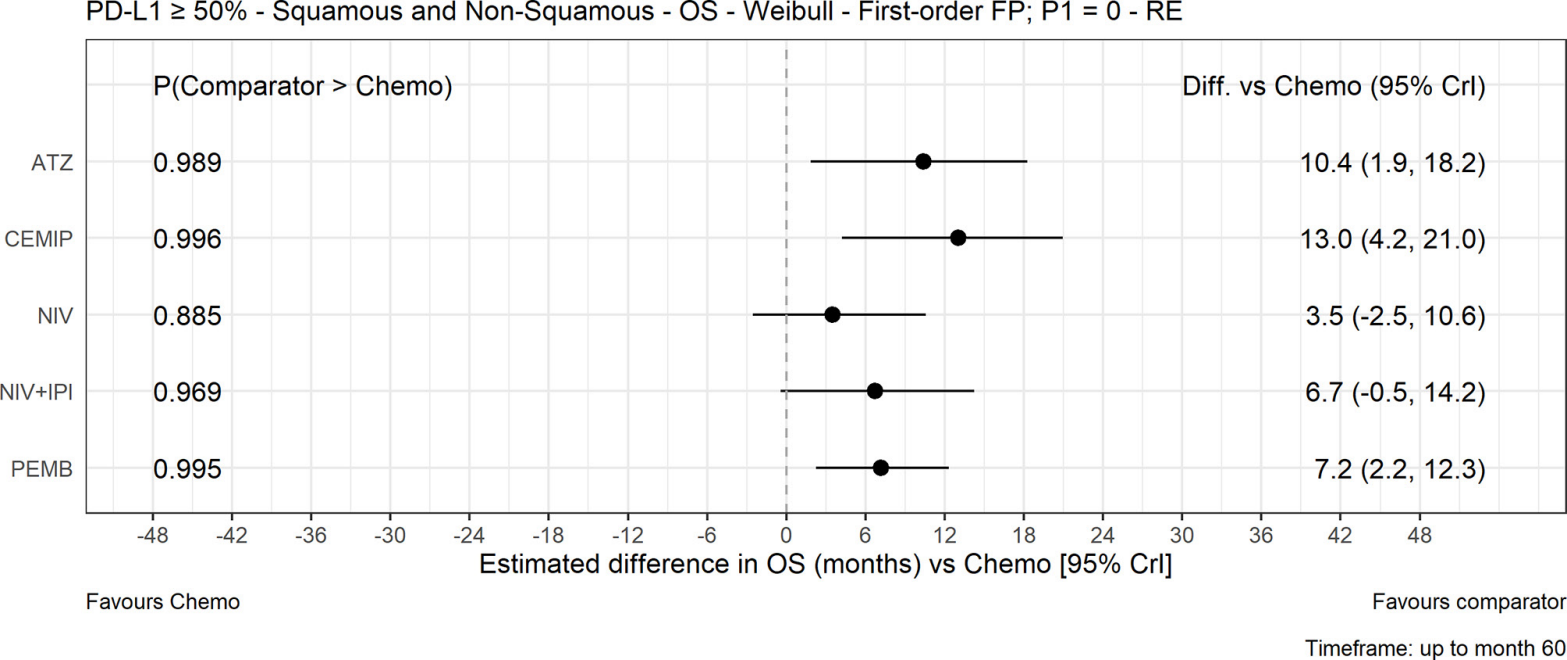
Expected OS difference with chemotherapy *versus* cancer immunotherapy comparators, mixed histology (60-month time horizon). Median posterior estimate and 95% posterior CrI. ATZ, atezolizumab; CEMIP, cemiplimab; chemo, chemotherapy; Diff., difference; NIV, nivolumab; NIV+IPI, nivolumab plus ipilimumab; OS, overall survival; PD-L1, programmed death-ligand 1; PEMB, pembrolizumab; FP, fractional polynomial; P1=0, Weibull; RE, random effects.

**Figure 3:**
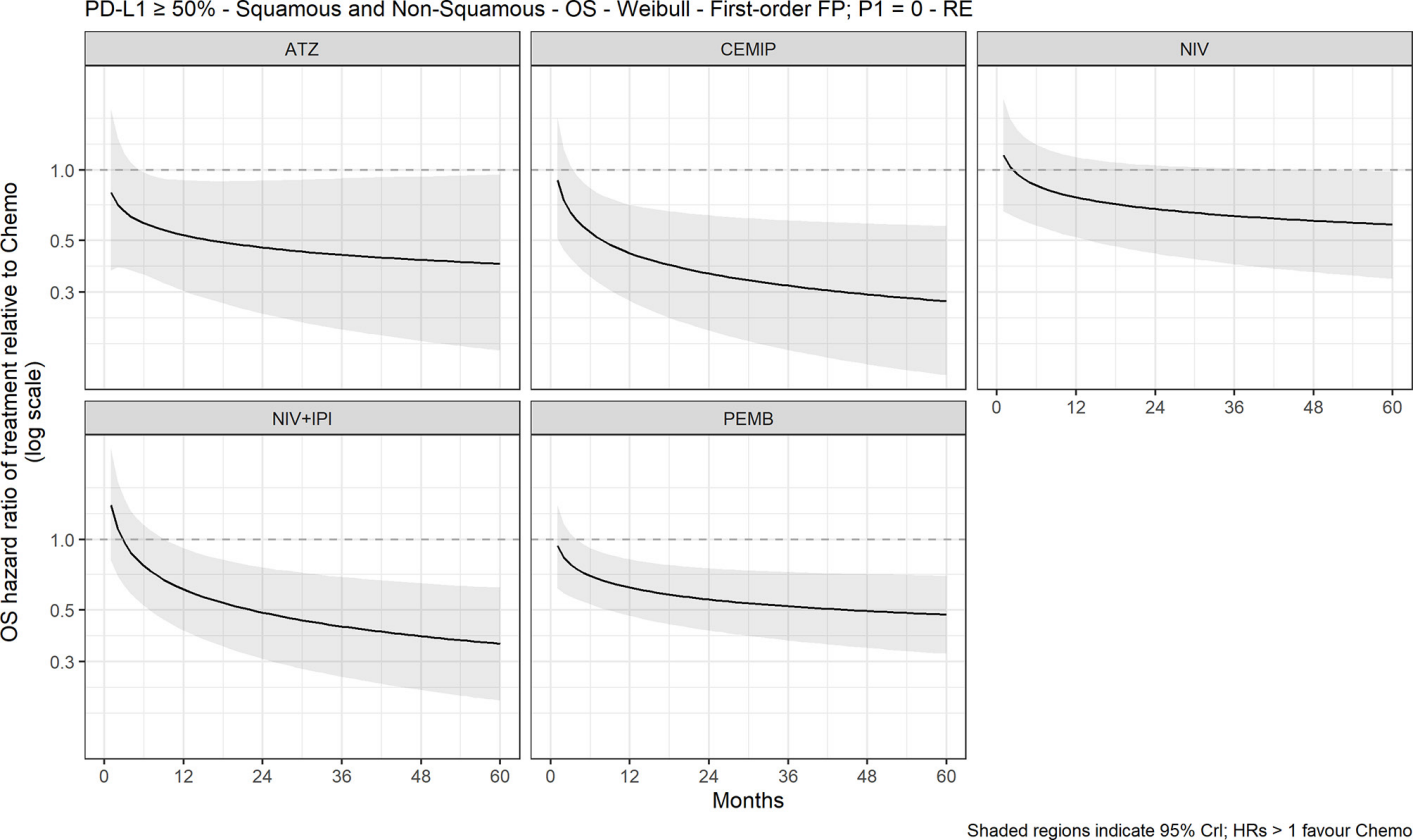
Expected OS HR over time for cancer immunotherapy *versus* chemotherapy with 95% CrI, mixed histology (60-month time horizon). ATZ, atezolizumab; CEMIP, cemiplimab; chemo, chemotherapy; Diff., difference; HR, hazard ratio; NIV, nivolumab; NIV+IPI, nivolumab plus ipilimumab; OS, overall survival; PD-L1, programmed death-ligand 1; PEMB, pembrolizumab; FP, fractional polynomial, P1=0, Weibull. RE, random effects.

The FP NMA models for OS fit the data reasonably well for all treatments and studies. In the mixed-histology network of PD-L1–high patients, the expected OS was significantly longer with atezolizumab (10.4 months [95% CrI: 1.9, 18.2]), pembrolizumab (7.2 months [2.2, 12.3]), and cemiplimab (13.0 [4.2, 21.0]) *versus* chemotherapy but not with nivolumab or nivolumab plus ipilimumab *versus* chemotherapy (**Figure 2**). The OS HRs over time illustrated the delayed onset of treatment effect observed with CIT, where all regimens, except nivolumab, appeared to show superiority *versus* chemotherapy (**Figure 3**) from approximately 9 months onward. Results were consistent in the NMA based on HRs with proportional hazards assumptions, where atezolizumab (HR, 0.59 [95% CrI: 0.37, 0.95]), pembrolizumab (0.66 [0.52, 0.85]), and cemiplimab (0.57 [0.38, 0.85]) showed significantly improved survival compared with chemotherapy; the durvalumab and nivolumab regimens showed lower HRs but were not significant compared with chemotherapy (**Figure S2**).

In the squamous-histology network, OS was not significantly longer with any of the treatment regimens *versus* chemotherapy, including pembrolizumab with carboplatin and paclitaxel (CP; 2.5 months [−4.7, 17.0]), atezolizumab (14.5 months [−1.4, 29.4]), or atezolizumab with CP (7.2 months [−1.3, 18.6]; **Figure S3**). In the proportional hazards analysis, pembrolizumab showed significantly improved survival *versus* chemotherapy (HR, 0.58 [0.39, 0.89]; **Figure S3**).

In the non–squamous histology network, the pembrolizumab plus platinum-based chemotherapy followed by maintenance pemetrexed regimen significantly improved survival compared with chemotherapy in both the FP NMA (8.9 months [0.4, 18.3]) and the proportional hazards HR analysis (HR, 0.59 [0.35, 0.99]; **Figure S4**). Pembrolizumab monotherapy also significantly improved survival compared with chemotherapy in the proportional hazards HR analysis (HR, 0.71 [0.51, 0.97]).

### Progression-Free Survival

**Figure 4:**
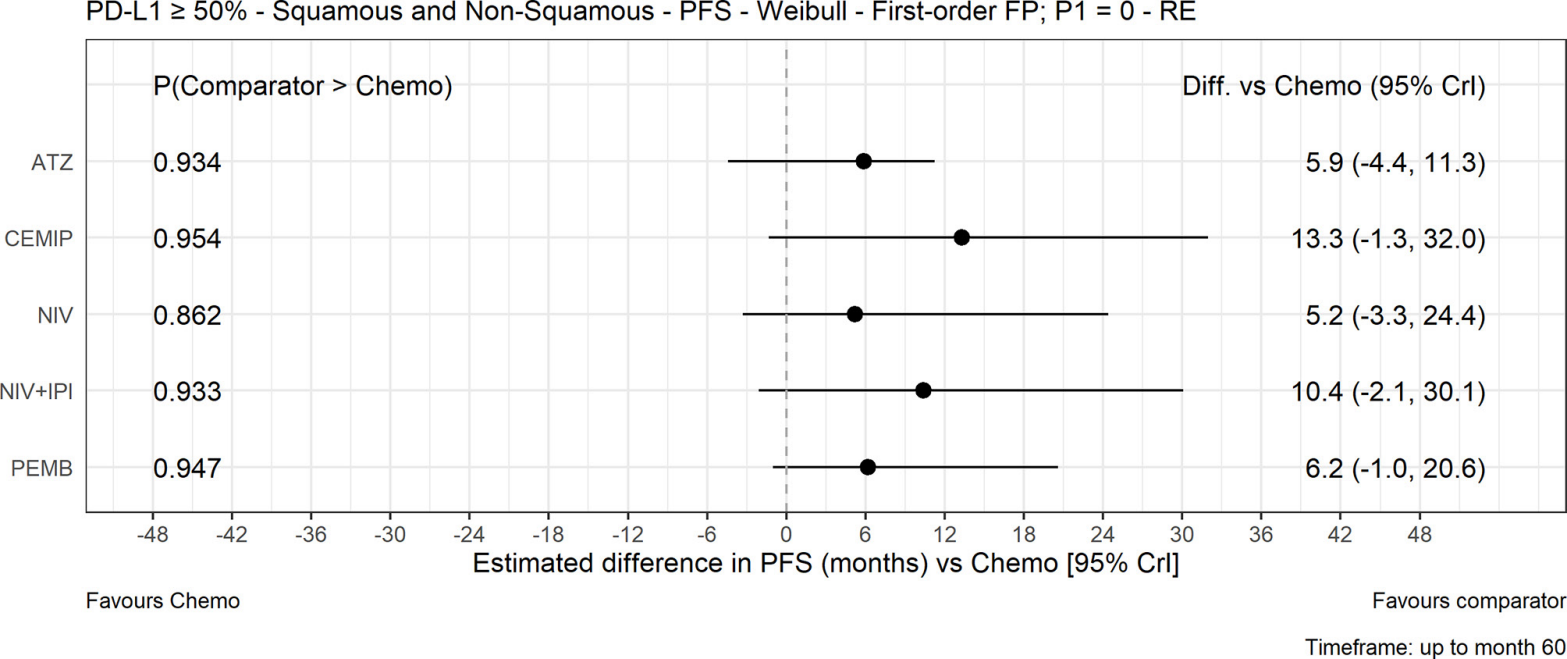
Expected PFS difference with chemotherapy *versus* cancer immunotherapy comparators, mixed histology (60-month time horizon). Median posterior estimate and 95% posterior CrI. ATZ, atezolizumab; CEMIP, cemiplimab; chemo, chemotherapy; Diff., difference; NIV, nivolumab; NIV+IPI, nivolumab plus ipilimumab; PD-L1, programmed death-ligand 1; PFS, progression-free survival; PEMB, pembrolizumab; FP, fractional polynomial; P1=0, Weibull; RE, random effects.

**Figure 5:**
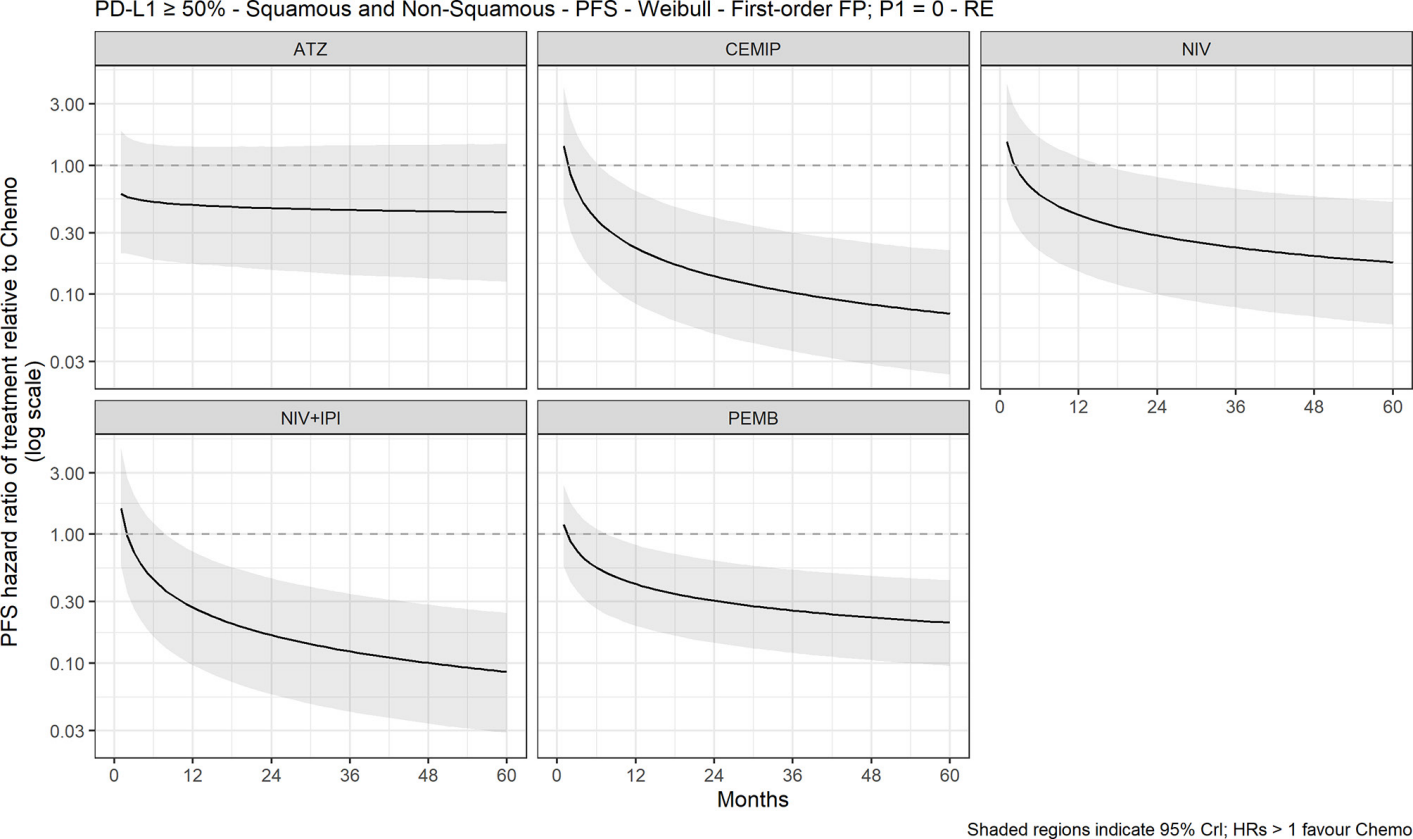
Expected PFS HR over time for cancer immunotherapy *versus* chemotherapy with 95% CrI, mixed histology (60-month time horizon). ATZ, atezolizumab; CEMIP, cemiplimab; HR, hazard ratio; NIV, nivolumab; NIV+IPI, nivolumab plus ipilimumab; PD-L1, programmed death-ligand 1; PEMB, pembrolizumab; PFS, progression-free survival; FP, fractional polynomial; P1=0, Weibull; RE, random effects.

The FP NMA models for PFS fit the data reasonably well for all treatments and studies. There is a marginally poorer fit to the atezolizumab data where PFS is slightly overestimated at the start of the follow-up period and to the KEYNOTE-024 data where PFS is overestimated at first then underestimated later in the follow-up period in both arms. In the mixed-histology evidence network, PFS increase was not significant with any of the CIT regimens *versus* chemotherapy in both the FP NMA (**Figure 4**) and proportional hazards HR analysis (**Figure S5**). The analysis of HRs over time showed that the improvement in PFS was not significant over 60 months with atezolizumab, while for pembrolizumab, cemiplimab, nivolumab plus ipilimumab, and nivolumab monotherapy, it became significant over time (**Figure 5**).

PFS increase was not significant with any of the CIT treatment regimens *versus* chemotherapy in the FP NMA or proportional hazards HR squamous-histology networks (**Figure S6**). All CIT regimens included in the non-squamous network significantly improved PFS compared with chemotherapy in the FP NMA, including atezolizumab monotherapy, atezolizumab plus CP, atezolizumab plus CP and bevacizumab (CPB), pembrolizumab plus C followed by maintenance pemetrexed, and sintilimab plus pemetrexed and platinum-based chemotherapy (**Figure S7**). All CIT regimens, including tislelizumab plus chemotherapy, significantly improved PFS compared with chemotherapy in the non-squamous proportional hazards model analysis, with the exception of atezolizumab monotherapy, for which the upper CrI lies just above 1.0 (HR, 0.55 [0.30, 1.01]; **Figure S7**).

### Objective Response Rate

In the mixed-histology network NMA analysis of ORR, pembrolizumab monotherapy (odds ratio [OR], 1.55 [1.10, 2.29]) and cemiplimab monotherapy (OR, 2.55 [1.52, 4.24]) significantly improved ORR compared with chemotherapy (**Figure S8**). In the squamous network, CP combination regimens with atezolizumab (OR, 3.12 [1.08, 9.15]) and pembrolizumab (3.07 [1.24, 8.18]), except with atezolizumab plus CP and bevacizumab, significantly improved ORR compared with chemotherapy, as did atezolizumab plus CP (OR, 2.41 [1.17, 5.02]), pembrolizumab plus platinum-based chemotherapy followed by maintenance pemetrexed (OR, 5.85 [3.05, 11.90]), and sintilimab (OR, 3.45 [1.61, 7.25]) in the non-squamous network (**Figure S8**).

### Safety

In the mixed-histology network, the CIT regimens with evaluable safety data (atezolizumab and pembrolizumab) had significantly lower odds of any TRAE than chemotherapy (**Figure S9**), but there was no significant difference between the CIT regimens and chemotherapy.

## Discussion

This NMA synthesized all available direct and indirect evidence of CIT regimens *versus* chemotherapy for first-line NSCLC treatment. In the mixed-histology network FP NMA, OS estimates were significantly longer with the atezolizumab and pembrolizumab monotherapy regimens—but not with the nivolumab or nivolumab plus ipilimumab regimens—compared with chemotherapy. None of the CIT regimens were significantly different from chemotherapy in either the squamous or non-squamous NMAs for OS, with the exception of pembrolizumab plus platinum-based chemotherapy followed by maintenance pemetrexed for non-squamous NSCLC. The analysis of OS HRs over time illustrated the delayed treatment effect of CIT compared with chemotherapy. None of the CIT regimens showed significant advantages in PFS *versus* chemotherapy for the 60-month time horizon FP NMA analysis in the mixed- or squamous-histology networks. Expected PFS differences were greater with the CIT regimens in the non-squamous network, which included atezolizumab and sintilimab monotherapy regimens, and atezolizumab or pembrolizumab combination regimens. ORR was significantly better with pembrolizumab and cemiplimab monotherapy regimens *versus* chemotherapy in the mixed-histology network and with carboplatin and paclitaxel combinations with atezolizumab or pembrolizumab in the squamous-histology network. ORR was also significantly better with sintilimab, atezolizumab, or pembrolizumab combination regimens in the non-squamous FP NMA network. Chemotherapy showed greater odds of any TRAE compared with the CIT regimens with evaluable safety data. Our findings suggest that monotherapy or combination regimens with atezolizumab or pembrolizumab offer greater OS and ORR benefits with less risk of side effects than a traditional chemotherapy regimen alone. Our findings are confirmatory of the individual trial findings for CIT *versus* chemotherapy and are consistent with NCCN and ESMO recommendations for first-line treatment of patients with NSCLC and high PD-L1 expression.

NMA is a useful approach to synthesize direct and indirect evidence of available treatments when patient populations and outcome measures may be appropriately aggregated. Our NMA of CIT and chemotherapy used the most recent clinical trial findings and the most appropriate statistical methods accounting for CIT-specific considerations. An indirect comparison analysis was required because of the absence of direct comparison trials. Liu (9) compared atezolizumab- and pembrolizumab-containing CIT regimens across histology and PD-L1 expression subgroups using the IMpower and KEYNOTE trial results available at the time (9). Our findings were generally consistent with those of Liu et al. (9), who reported favorable results with CIT-containing regimens, with some differences—such as improved outcomes when bevacizumab was combined with atezolizumab and chemotherapy. Tun (10) showed improved OS, PFS, and ORR when adding CIT to chemotherapy for first-line NSCLC treatment, even among patients with *EGFR* alterations and *ALK* translocations, with incremental increase in adverse events (10). Meta-analyses by Addeo (39) and Chen (11) suggested improved OS, PFS, and ORR with CIT with or without chemotherapy *versus* chemotherapy but did not use a Bayesian FP NMA approach and did not include the IMpower110 trial findings. Their results were more favorable for subgroups with high PD-L1 expression (11, 39), which was the focus of our FP NMA.

This NMA should be interpreted in the context of certain strengths and limitations. Individual patient data were only available for the atezolizumab trials; digitized summary data extracted from publications were used for other trials. Findings were consistent across histology subgroups and sensitivity analyses that included different contributing clinical trials for each of the CIT interventions. In general, heterogeneity was low within the evidence networks where evidence was available for assessment. One exception was noted with the PFS comparison of pembrolizumab and chemotherapy in the squamous and non-squamous networks (KEYNOTE-024, KEYNOTE-042). Many treatment comparisons were informed by a single study, limiting the means to quantitatively evaluate between-study heterogeneity. As such, informative priors for the between-study variance were used to fit random effects models. All chemotherapy treatment arms across trials were considered exchangeable and assigned to the same network node. This may have introduced heterogeneity in the analysis, resulting in undetected biases when estimating the different treatment effects. There were no closed loops in the networks, and inconsistency could not be evaluated. PFS data from the atezolizumab studies were based on investigator assessment, whereas all other studies used independent review committee assessments. We assumed these to be equivalent, but this may have been a source of inherent bias. Sub-populations for each study for every possible histology group in the PD-L1-high population were analyzed. The individual studies were not necessarily powered to inform these comparisons, and in some instances, these subgroups included few patients (e.g.., the squamous NSCLC PD-L1-high IMpower110 population included 50 patients across the two treatment groups). Length of follow-up was relatively short for some studies, which may be a partial reflection of the poor prognosis for advanced NSCLC and introduce uncertainty regarding longer-term effects. For some trials, updated findings based on longer follow-up times were not available for this report, as some data were from reports of interim analyses. Estimated quantities from the FP analysis, such as expected OS, expected PFS, hazard ratios, and survival functions over time, were presented for a period of 5 years maximum and should be interpreted with caution, in particular for the later time points. Heterogeneity of PD-L1 assay methods used across trials remains a point of concern when conducting indirect comparisons across CIT treatment options. Finally, although it was not an objective of this analysis, further work is needed to better understand the relative effectiveness of CIT for other PD-L1 expression subgroups.

## Conclusions

This systematic literature review and NMA suggested superiority of CIT regimens *versus* chemotherapy as first-line treatment of NSCLC in terms of survival, objective response, and safety. These findings may support evidence evaluations and decisions in the clinic and for health technology assessments applied to population health policies.

## Author Contributions

All authors contributed to the interpretation of results and development of the manuscript, including approval for submission. SA, DJ, RM, and RB designed the study. RM and DJ collected and analyzed the data. All authors contributed to the article and approved the submitted version.

## Funding

This study is sponsored by F. Hoffmann-La Roche, Ltd.

## Conflict of Interest

RH reports non-financial support from Genentech/Roche during the conduct of the study; personal fees from Abbvie Pharmaceuticals, personal fees from ARMO Biosciences, grants and personal fees from AstraZeneca, personal fees from Biodesix, personal fees from Bolt Biotherapeutics, personal fees from Bristol Myers Squibb, personal fees from Cybrexa Therapeutics, personal fees from eFFECTOR Therapeutics, Inc., grants and personal fees from Eli Lilly and Company, personal fees from EMD Serono, grants and personal fees from Genentech/Roche, personal fees from Genmab, personal fees from Halozyme Therapeutics, personal fees from Heat Biologics, personal fees from I-Mab Biopharma, personal fees from Immunocore, personal fees from Infinity Pharmaceuticals, personal fees from Junshi Pharmaceuticals, personal fees from Loxo Oncology, grants and personal fees from Merck and Company, personal fees from Mirati Therapeutics, personal fees from Nektar, personal fees from Neon Therapeutics, personal fees from NextCure, personal fees from Novartis, personal fees from Oncternal Therapeutics, personal fees from Pfizer, personal fees from Sanofi, personal fees from Seattle Genetics, personal fees from Shire PLC, personal fees from Spectrum Pharmaceuticals, personal fees from STCube Pharmaceuticals, Inc, personal fees from Symphogen, personal fees from Takeda, personal fees from Tesaro, personal fees from Tocagen, personal fees from WindMIL Therapeutics, personal fees from Xencor, Inc, outside the submitted work. JJ reports personal fees and non-financial support from Roche during the conduct of the study; personal fees from AstraZeneca, personal fees from Pfizer, personal fees from BMS, personal fees from MSD outside the submitted work. SA and RB are employees of F. Hoffmann-La Roche, Ltd, the study sponsor. DJ is an employee of Quantics Biostatistics, and RM is an employee of the York Health Economics Consortium, Ltd, both of which received funding from the study sponsor for this work. FM reports fees as advisor/speaker from AstraZeneca, BMS, Roche/Genentech, Novartis.

The remaining authors declare that the research was conducted in the absence of any commercial or financial relationships that could be construed as a potential conflict of interest.

The authors declare that this study received funding from F. Hoffmann-La Roche, Ltd. The funder was involved in the study design, collection and analysis of the data.
