# Supplementary material for: A Network Meta-Analysis of Cancer Immunotherapies Versus Chemotherapy for First-Line Treatment of Patients With Non-Small Cell Lung Cancer and High Programmed Death-Ligand 1 Expression
Source: Front Oncol. 2021 Jul 9;11:676732. doi: 10.3389/fonc.2021.676732 (PMC8300186; doi:10.3389/fonc.2021.676732)
Supplement: Supplementary file 1 [file DataSheet_1.pdf]

SUPPLEMENTARY MATERIAL

Table S1. Search terms

| Step | Search |
|---|---|
| 1 | Carcinoma, Non-Small-Cell Lung[mh:noexp] |
| 2 | (non small cell*[tiab] OR nonsmall cell*[tiab] OR large cell*[tiab] OR squamous cell*[tiab] OR squamous small cell*[tiab] OR nonsquamous cell*[tiab] OR nonsquamous small cell*[tiab] OR epidermoid[tiab]) AND (cancer*[tiab] OR carcin*[tiab] OR tumor*[tiab] OR tumour*[tiab] OR neoplas*[tiab] OR oncol*[tiab] OR malignan*[tiab]) |
| 3 | lung[tiab] OR lungs[tiab] OR pulmonary[tiab] OR bronchial OR bronchus[tiab] |
| 4 | 2 AND 3 |
| 5 | (lung[tiab] OR lungs[tiab] OR pulmonary[tiab] OR bronchial[tiab]) AND (adenocarcin*[tiab] OR adenocancer*[tiab]) |
| 6 | NSCLC*[tiab] |
| 7 | 1 OR 4 OR 5 OR 6 |
| 8 | randomized controlled trial[pt] |
| 9 | controlled clinical trial[pt] |
| 10 | randomized[tiab] |
| 11 | placebo[tiab] |
| 12 | clinical trials as topic[mh:noexp] |
| 13 | randomly[tiab] |
| 14 | trial[ti] |
| 15 | 8 OR 9 OR 10 OR 11 OR 12 OR 13 OR 14 |
| 16 | 7 AND 15 |
| 17 | Animals[mh] NOT Humans[mh:noexp] |
| 18 | news[pt] OR comment[pt] OR letter[pt] OR editorial[pt] OR case reports[pt] OR case report[ti] |
| 19 | 16 NOT (17 OR 18) |

This search strategy for PubMed/MEDLINE was adapted for Embase.

Table S2. Data sources searched

| Database/Information source | Interface/Address | Search Date |
|---|---|---|
| **Electronic Databases** | | |
| Cochrane Central Register of Controlled Trials (CENTRAL) | Cochrane Library/Wiley: https://www.cochranelibrary.com/ | 15 Sep 2020 |
| Cochrane Database of Systematic Reviews (CDSR) | Cochrane Library/Wiley: https://www.cochranelibrary.com/ | 15 Sep 2020 |
| Embase (including MEDLINE) | Embase.com | 16 Sep 2020 |
| Health Technology Assessment database (HTA) | Centre for Reviews and Dissemination: https://www.crd.york.ac.uk/CRDWeb/ | 16 Sep 2020 |
| PubMed | http://www.ncbi.nlm.nih.gov/pubmed | 16 Sep 2020 |
| **Study Registries and HTA websites** | | |
| ClinicalTrials.gov | https://www.clinicaltrials.gov/ct | 16 Sep 2020 |
| WHO International Clinical Trials Platform (ICTRP) | http://www.who.int/ictrp/en/ | 16 Sep 2020 |
| EU Clinical Trials Registry | https://www.clinicaltrialsregister.eu/ctr-search/search | 16 Sep 2020 |
| Drugs@FDA database | https://www.accessdata.fda.gov/scripts/cder/daf/index.cfm | 16 and 22 Sep 2020 |
| European Medicines Agency (EMA) medicines webpages | https://www.ema.europa.eu/en/medicines | 17 Sep 2020 |
| National Institute for Health and Care Excellence (NICE) webpages | https://www.nice.org.uk/ | 17 Sep 2020 |
| Canadian Agency for Drugs and Technologies in Health (CADTH) pan-Canadian Oncology Drug Review (pCODR) webpages | https://cadth.ca/pcodr | 17 Sep 2020 |
| Institut für Qualität und Wirtschaftlichkeit im Gesundheitswesen (IQWiG) webpages | https://www.iqwig.de/ | 22 Sep 2020 |
| **Conference Proceedings** | | |
| American Society of Clinical Oncology (ASCO) | Searched via the Embase database (if the conference was indexed in Embase) or via the conference website or journal webpages (if free to access) | 16 Sep 2020 |
| European Society for Medical Oncology (ESMO) | Searched via the Embase database (if the conference was indexed in Embase) or via the conference website or journal webpages (if free to access) | 21 Sep 2020 and 5 Oct 2020 |
| International Association for the Study of Lung Cancer (IASLC) | Searched via the Embase database (if the conference was indexed in Embase) or via the conference website or journal webpages (if free to access) | 11 Oct 2019 |
| World Conference on Lung Cancer (WCLC) | Searched via the Embase database (if the conference was indexed in Embase) or via the conference website or journal webpages (if free to access) | 11 Oct 2019 |

| | | |
|---|---|---|
| European Lung Cancer Conference (ELCC) | Searched via the Embase database (if the conference was indexed in Embase) or via the conference website or journal webpages (if free to access) | 11 Oct 2019 |
| British Thoracic Oncology Group (BTOG) conference | Searched via the Embase database (if the conference was indexed in Embase) or via the conference website or journal webpages (if free to access) | 16 Sep 2020 |

Table S3. Data extraction elements

| Grouping | Elements |
|---|---|
| Study details | Reference citation |
| Study characteristics | Study design<br>Study objective<br>Number of participating centers and countries<br>Eligibility criteria<br>Number of patients randomized/analyzed per outcome<br>Treatment and follow-up durations<br>Data collection time points<br>Statistical methods |
| Patient characteristics | Age<br>Gender<br>Time since diagnosis<br>Smoking status<br>Eastern Cooperative Oncology Group performance status<br>Ethnicity<br>Liver metastases<br>Previous treatments<br>EGFR mutation<br>ALK rearrangement<br>KRAS mutation<br>PD-L1 expression<br>Histology |
| Intervention(s) | Treatment<br>Dose<br>Regimen<br>Duration of treatment<br>Treatment interruptions and dosing modifications |
| Outcome(s) | Unit of measurement<br>Number of patients included in the analysis<br>Effect size<br>For dichotomous outcomes: absolute and relative risks (or odds ratios) and risk (or rate) differences<br>For continuous outcomes: the mean change and measure of variance from baseline (or at both baseline and final visit), or mean difference between treatments<br>For time-to-event analysis: the number of events in each arm, median time to event, and a hazard ratio and *p* value; Kaplan Meier curves were digitized where available<br>Where possible, absolute and relative data were extracted<br>A measure of precision for each estimate of effect (95% confidence intervals, standard error, or standard deviation) |

Table S4. Risk of bias assessment results

| Study | Was the allocation sequence adequately generated? | Was the concealment of treatment allocation adequate? | Was knowledge of the allocated interventions adequately prevented from participants and personnel | Was knowledge of the allocated interventions adequately prevented from outcome assessors | Were incomplete outcome data adequately addressed? | Are reports of the study free of suggestion of selective outcome reporting? | Was the study apparently free of other problems that could put it at a high risk of bias? |
|---|---|---|---|---|---|---|---|
| IMpower110 | Yes | Yes | No (open label) | Unclear (open label) | Yes | Yes | Yes |
| IMpower150 | Yes | Yes | No (open label) | Unclear (open label) | Yes | Yes | Yes |
| IMpower130 | Yes | Yes | No (open label) | Unclear | Yes | Yes | No |
| IMpower131 | Unclear | Unclear | No (open label) | Unclear | Unclear | Unclear | Yes |
| IMpower132 | Unclear | Unclear | No (open label) | Unclear | Unclear | Unclear | Yes |
| KEYNOTE-021 | Yes | Yes | No (open label) | Yes (open label)[a] | Yes | Yes | Yes |
| KEYNOTE-024 | Yes | Yes | No (open label) | Yes (open label)[a] | Yes | Yes | Yes |
| KEYNOTE-042 | Yes | Yes | No (open label) | Yes (open label)[a] | Yes | Yes | Yes |
| KEYNOTE-189 | Yes | Yes | Yes | Yes | Yes | Yes | Yes |
| KEYNOTE-407 | Yes | Yes | Yes | Yes | Yes | Yes | Unclear |
| CHECKMATE-026 | Yes | Unclear | No (open label) | Yes (open label)[a] | Yes | Yes | Yes |
| CHECKMATE-227 | Yes | Yes | No (open label) | Yes (open label)[a] | Yes | Yes | Yes |
| RATIONALE 304 | Unclear | Unclear | No (open label) | Unclear (open label) | Unclear | No | No |
| EMPOWER-LUNG 1 | Unclear | Unclear | No (open label) | Yes (open label)[a] | Unclear | Yes | No |
| MYSTIC | Yes | Yes | No (open label) | Yes (open label)[a] | Unclear | Yes | Yes |
| ORIENT-11 | Yes | Yes | Yes | Yes (open label)[a] | Yes | Yes | Yes |

[a] Open label trial (patients and personnel not blinded) but outcome assessors were reported to have been blinded.

Figure S1. Network diagrams

**(A)** Mixed-histology network

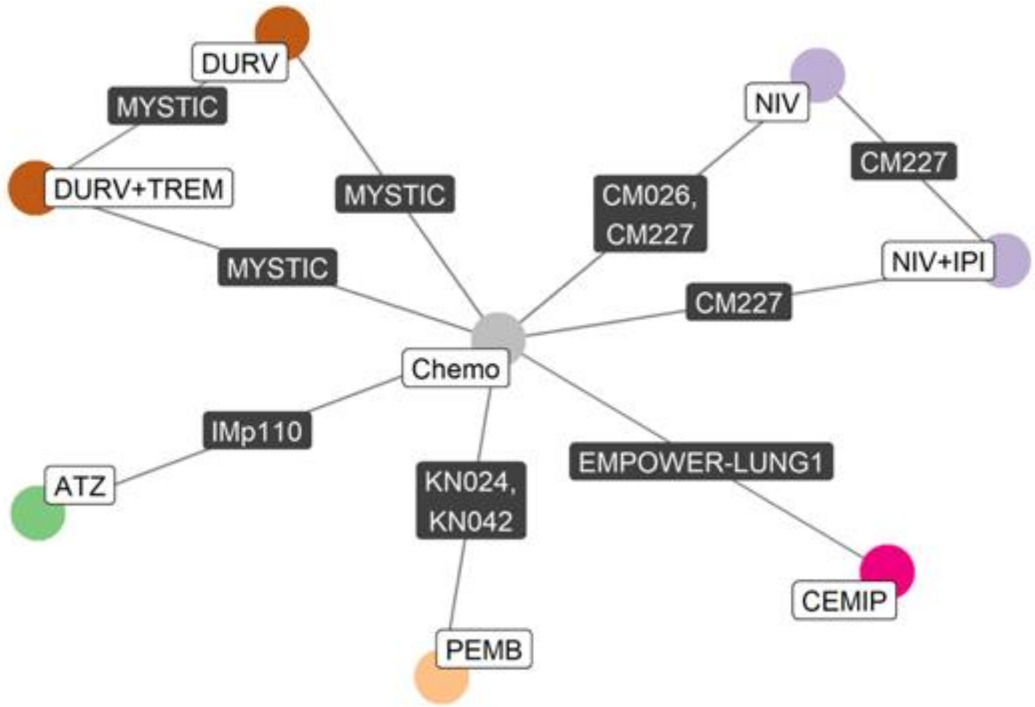


**(B)** Squamous histology network

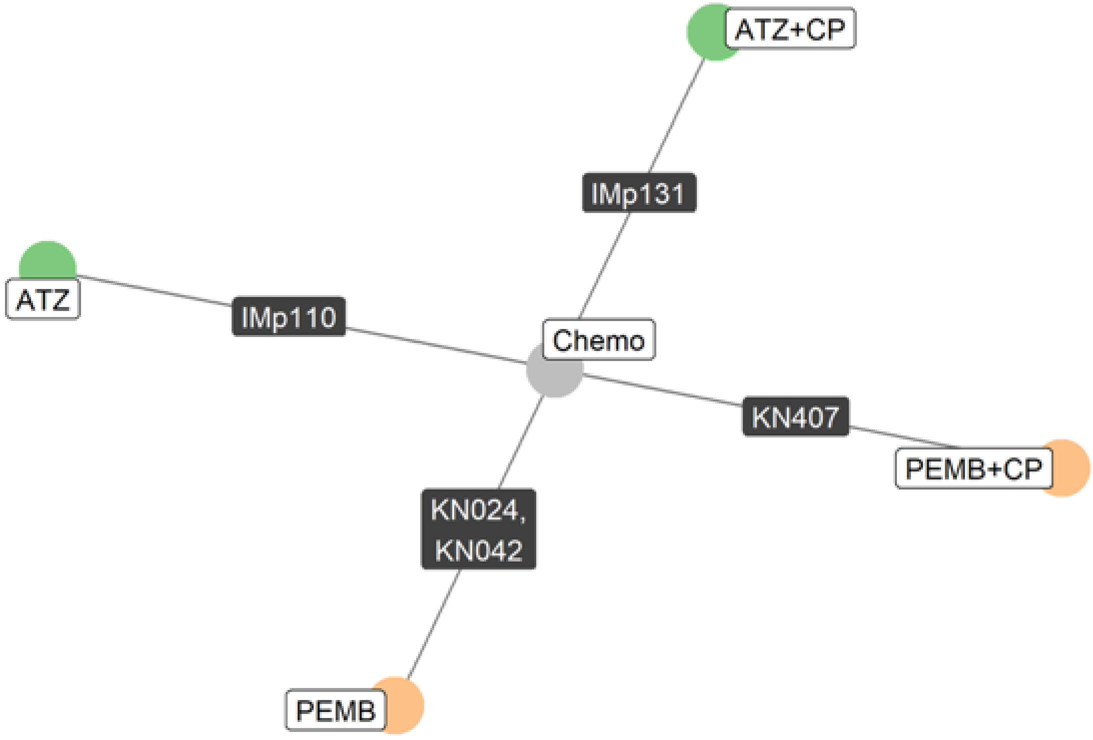

**(C)** Non-squamous histology network

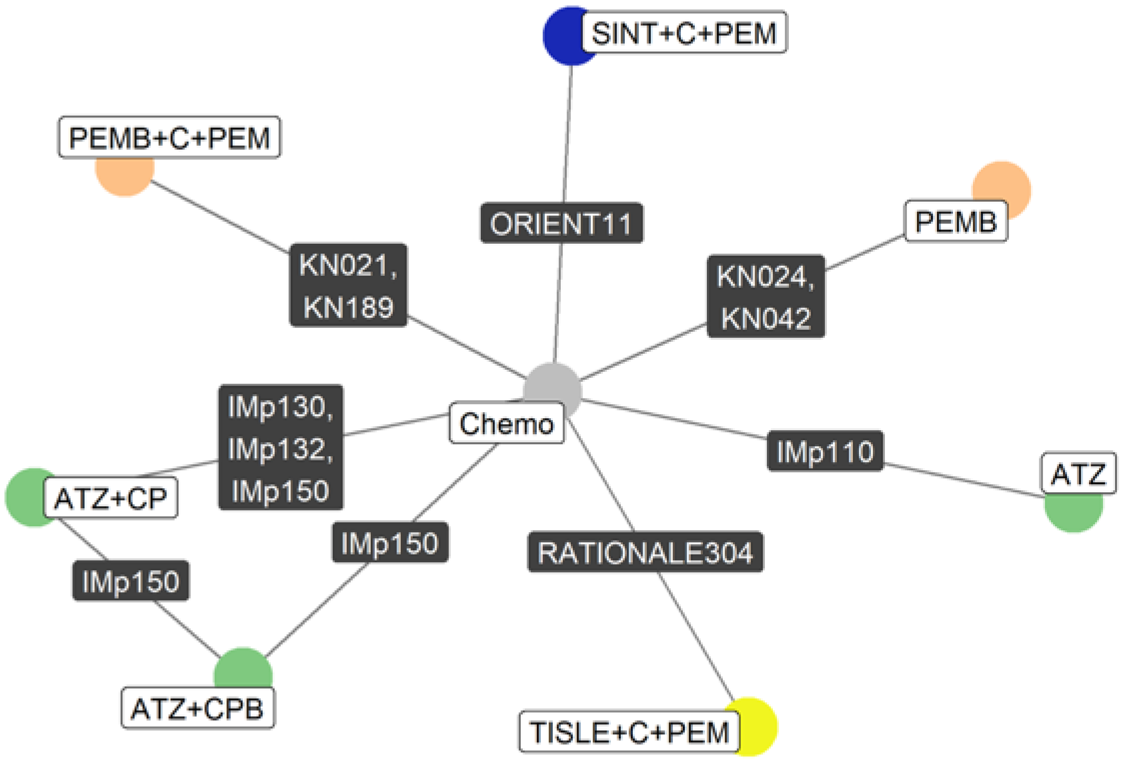


ATZ, atezolizumab; C, platinum-based chemotherapy; CEMIP, cemiplimab; CP, carboplatin plus paclitaxel; CPB, carboplatin plus paclitaxel plus bevacizumab; DURV, durvalumab; NIV, nivolumab; NIV+IPI, nivolumab plus ipilimumab; PEM, pemetrexed; PEMB, pembrolizumab; SINT, sintilimab; TISLE, tislelizumab; TREM, tremelimumab.

Figure S2. Mixed-histology non-small cell lung cancer: overall survival proportional hazards random effects model

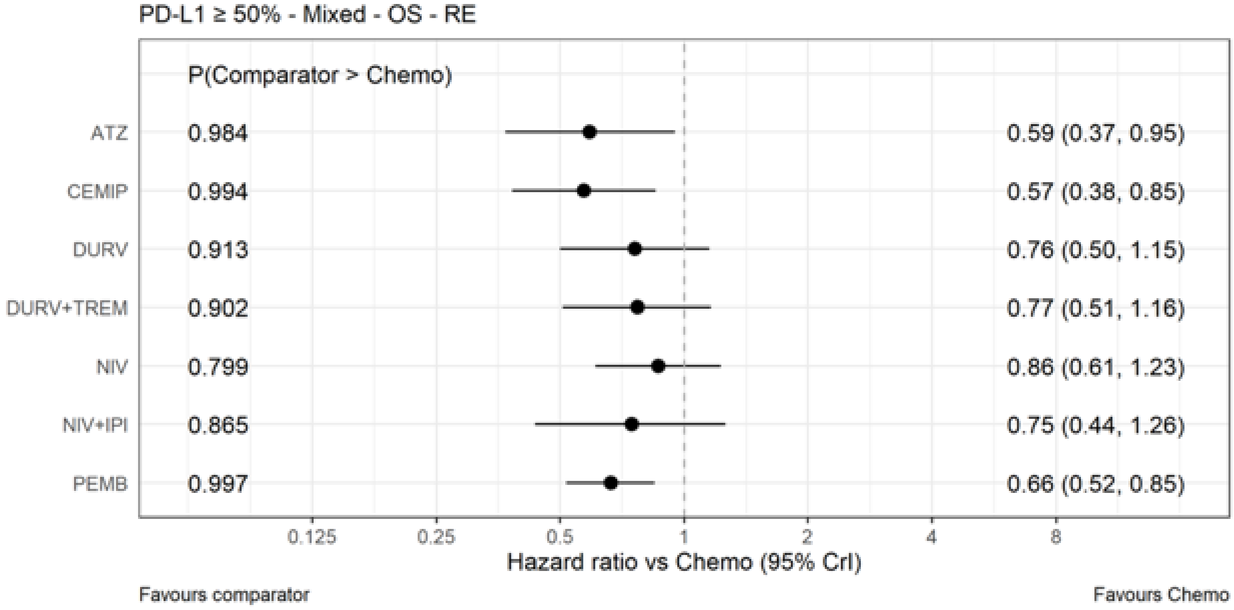


ATZ, atezolizumab; CEMIP, cemiplimab; DURV, durvalumab; NIV, nivolumab; NIV+IPI, nivolumab plus ipilimumab; PEMB, pembrolizumab; TREM, tremelimumab.

Figure S3. Squamous non-small cell lung cancer: OS FP, HRs over time, and proportional hazards HR analysis

**(A)** OS FP NMA model

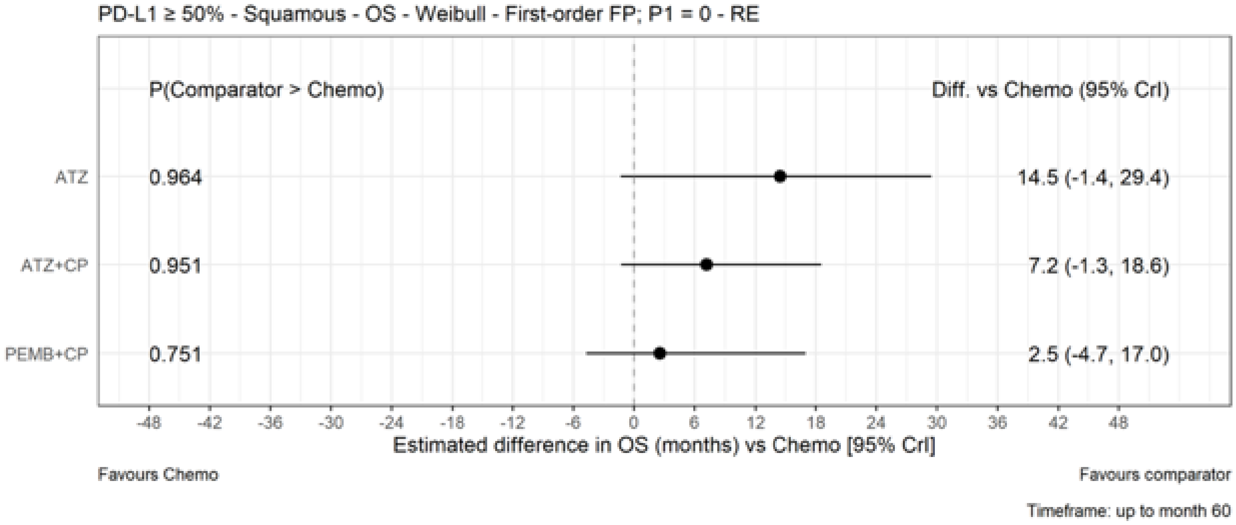


**(B)** Analysis of HRs over time

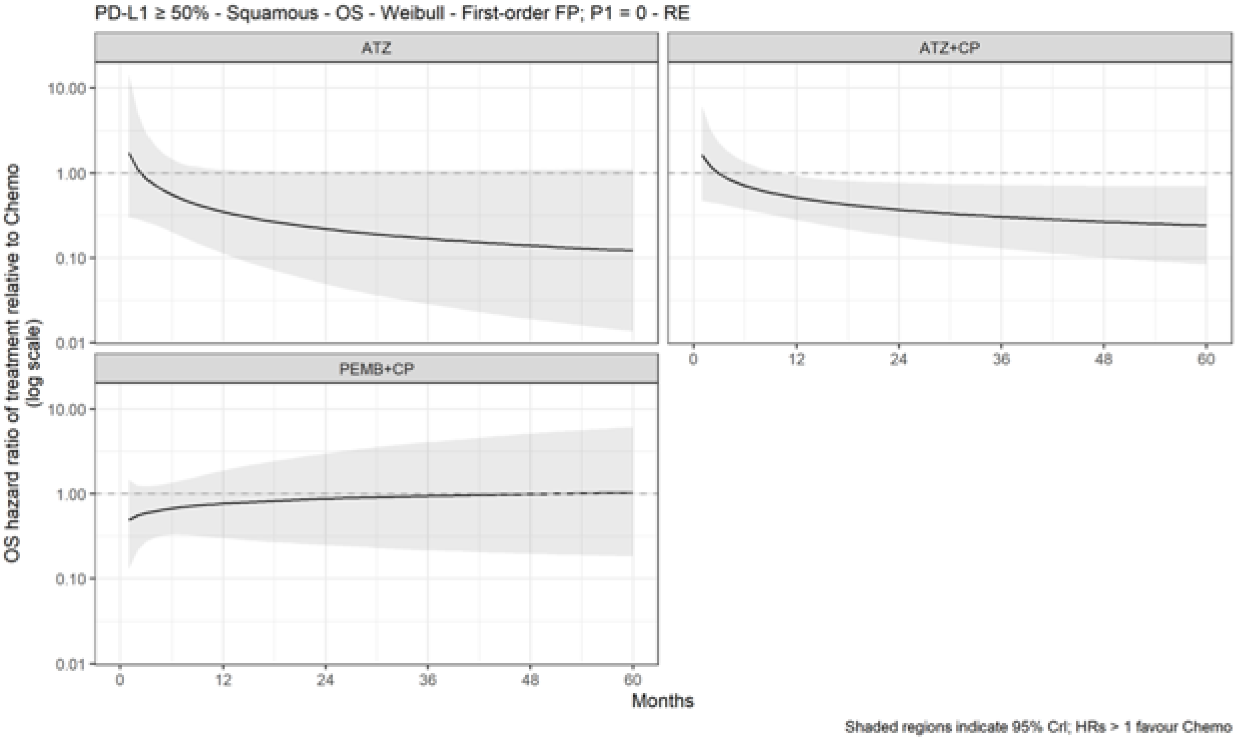

**(C)** OS proportional hazards random effects model

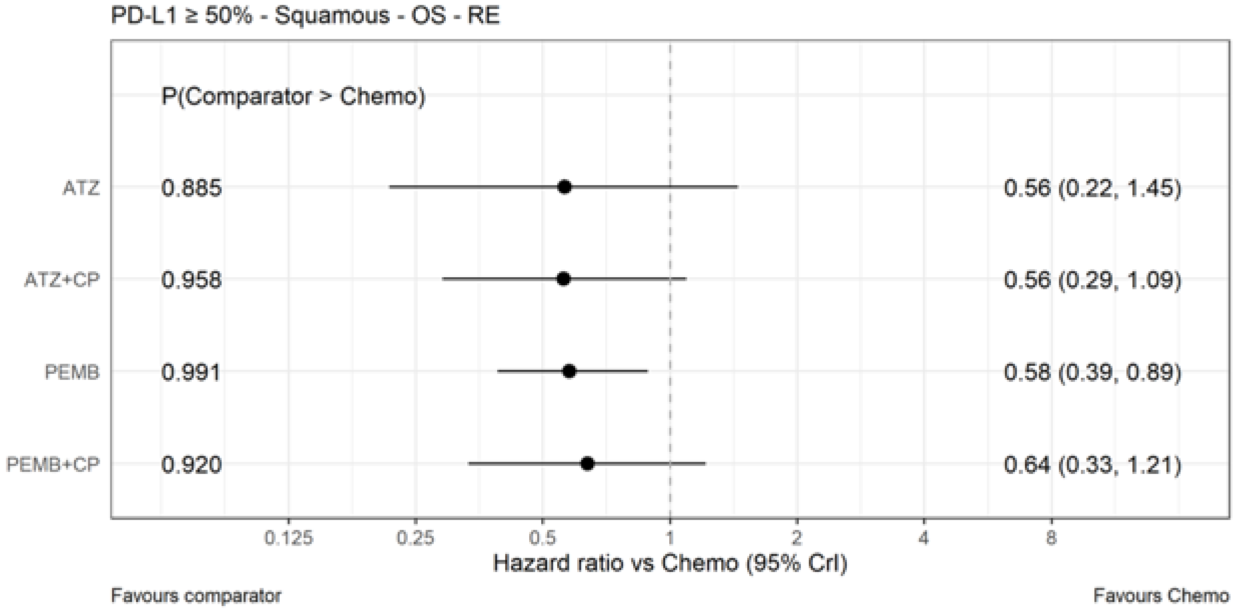


ATZ, atezolizumab; chemo, chemotherapy; Diff., difference; CP, carboplatin plus paclitaxel; HR, hazard ratio; FP, fractional polynomial; OS, overall survival; PD-L1, programmed death-ligand 1; PEMB, pembrolizumab.

Figure S4. Non-squamous non-small cell lung cancer: OS FP, HRs over time, and proportional hazards HR analysis

**(A)** OS FP NMA model

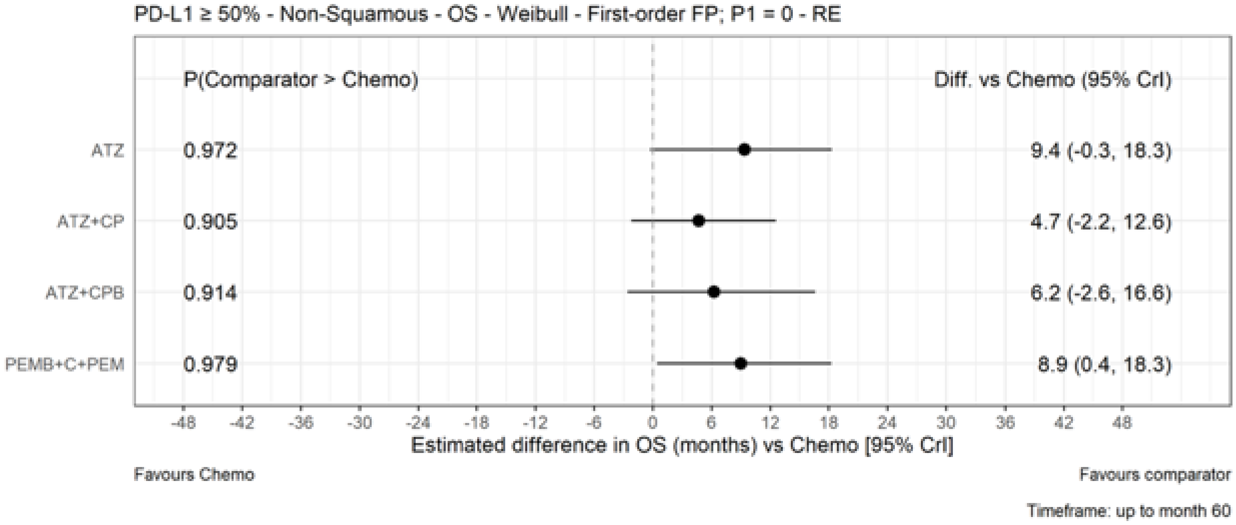


**(B)** Analysis of HRs over time

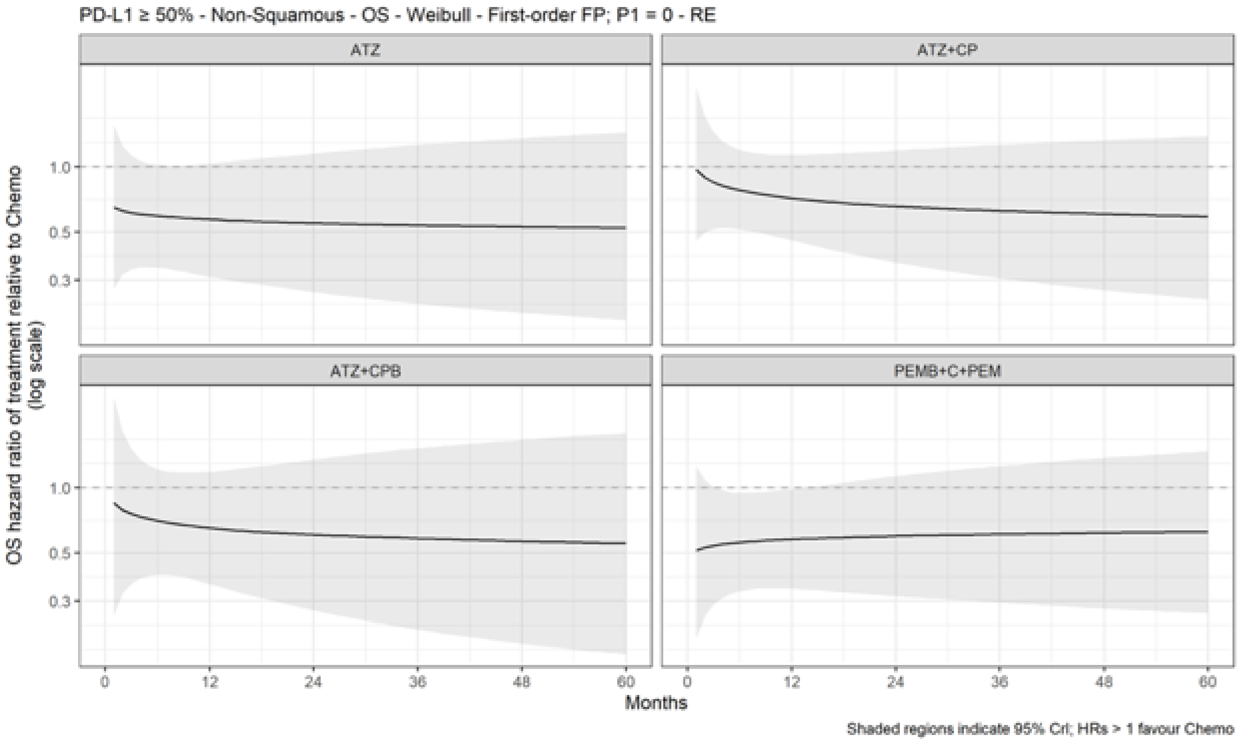

**(C)** OS proportional hazards random effects model

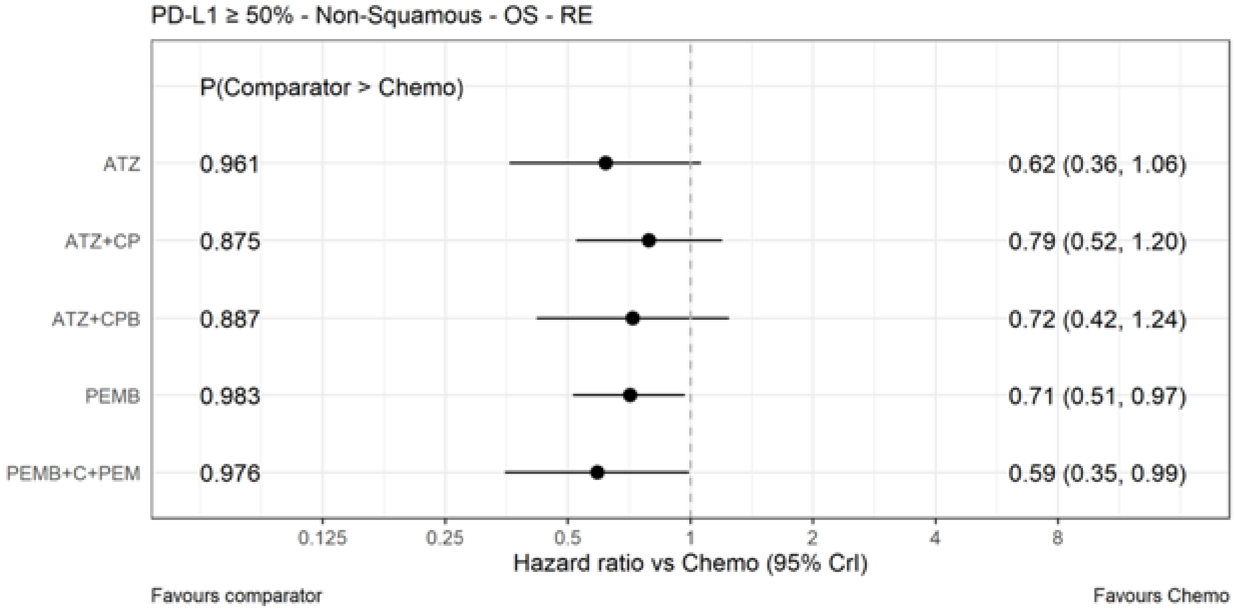


ATZ, atezolizumab; C, platinum-based chemotherapy; chemo, chemotherapy; CP, carboplatin plus paclitaxel; CPB, carboplatin plus paclitaxel plus bevacizumab; Diff., difference; FP, fractional polynomial; HR, hazard ratio; OS, overall survival; PD-L1, programmed death-ligand 1; PEM, pemetrexed; PEMB, pembrolizumab.

Figure S5. Mixed-histology non-small cell lung cancer: progression-free survival proportional hazards random effects model

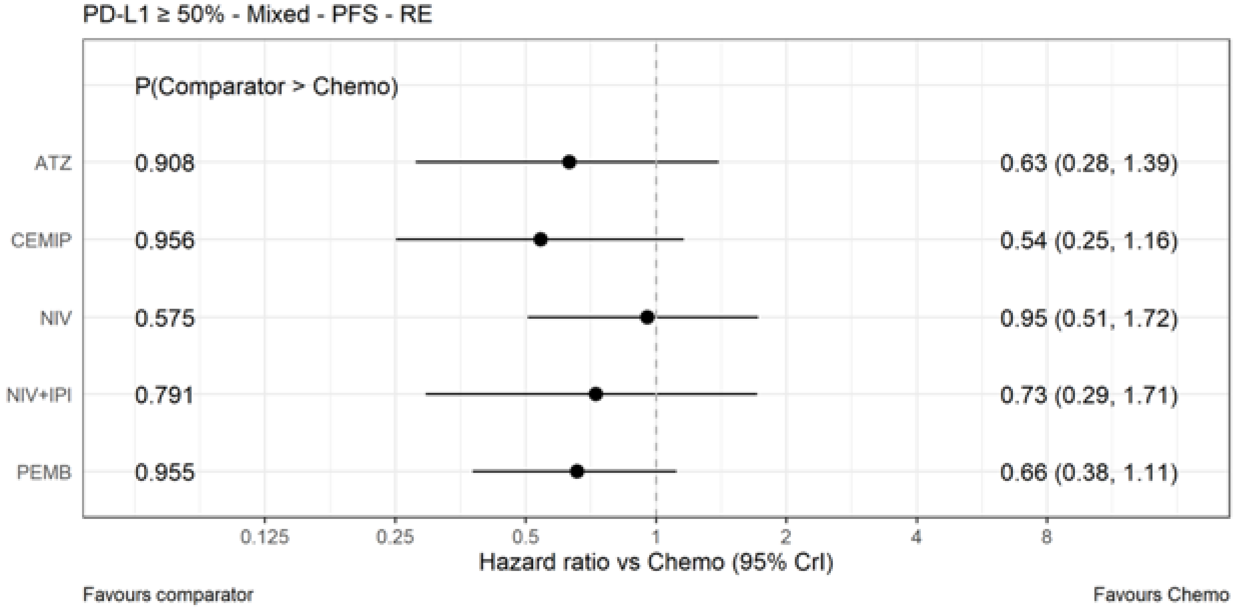


ATZ, atezolizumab; CEMIP, cemiplimab; NIV, nivolumab; NIV+IPI, nivolumab plus ipilimumab; PD-L1, programmed death-ligand 1; PEMB, pembrolizumab.

Figure S6. Squamous non-small cell lung cancer: PFS FP, HRs over time, and proportional hazards HR analysis

**(A)** PFS FP NMA model

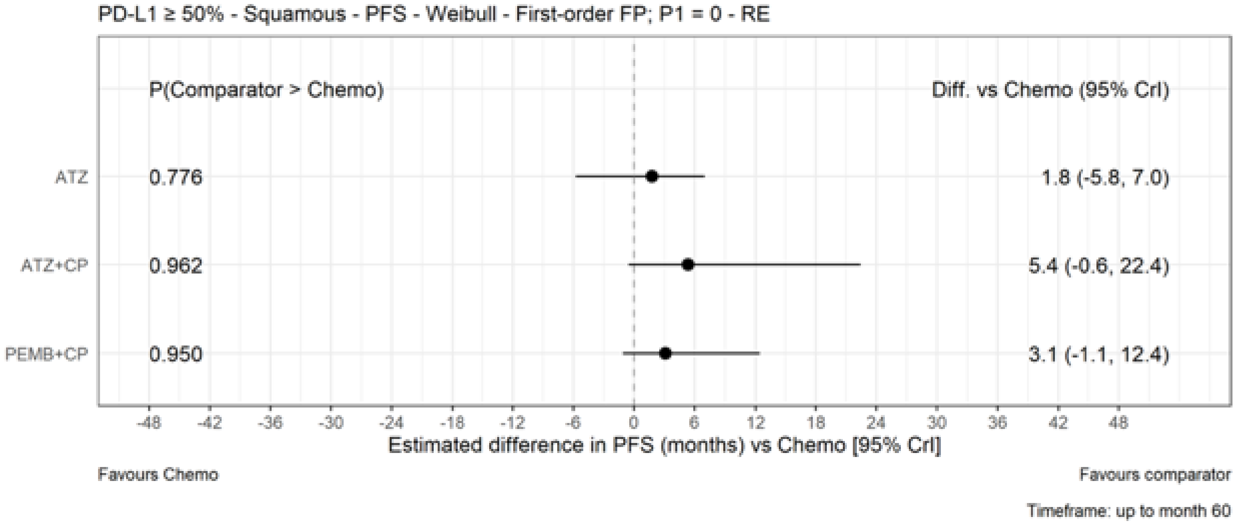


**(B)** Analysis of HRs over time

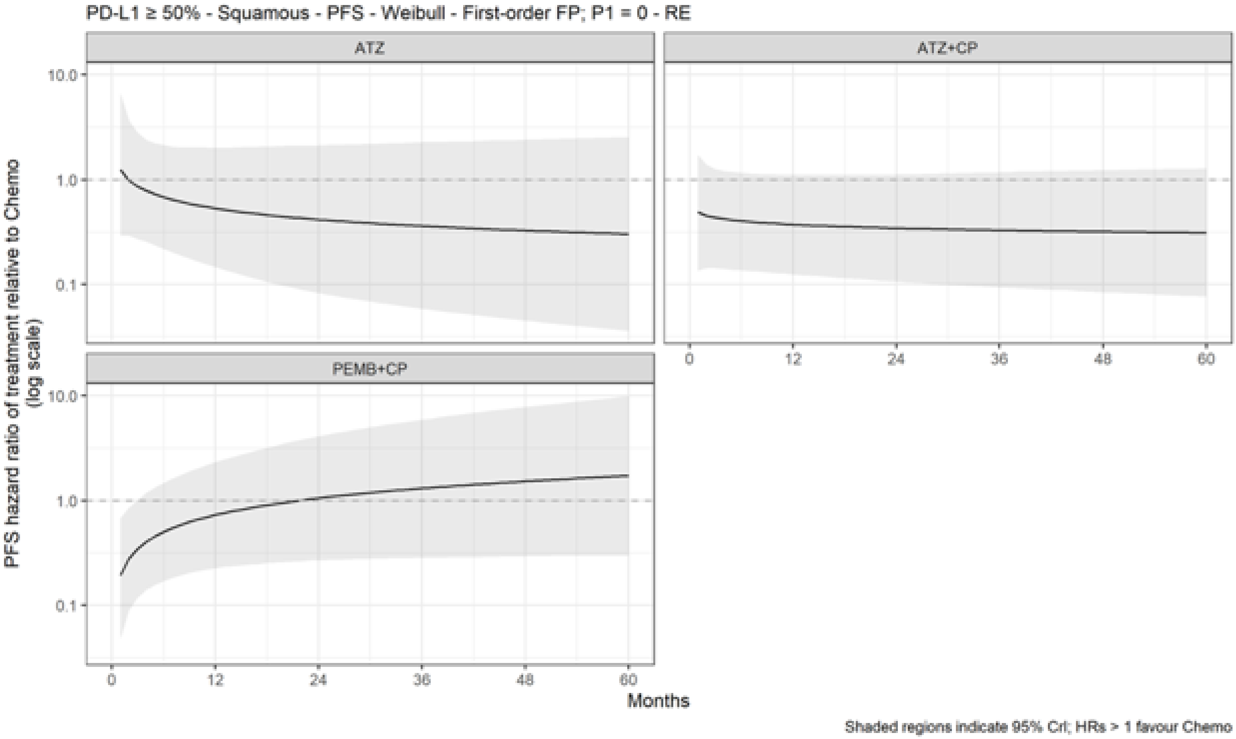

**(C)** PFS proportional hazards random effects model

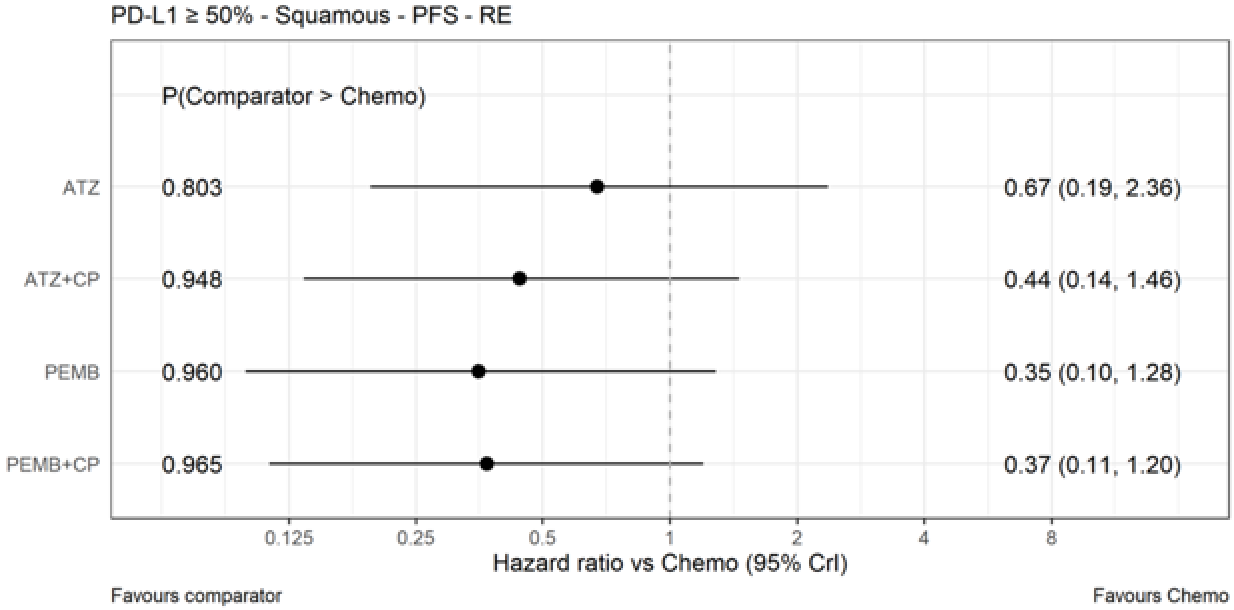


ATZ, atezolizumab; chemo, chemotherapy; chemo, chemotherapy; CP, carboplatin plus paclitaxel; Diff., difference; FP, fractional polynomial; HR, hazard ratio; PD-L1, programmed death-ligand 1; PEMB, pembrolizumab; PFS, progression-free survival.

Figure S7. Non-squamous non-small cell lung cancer: PFS, HRs over time and proportional hazards HR analysis

**(A)** PFS FP NMA model: estimated difference

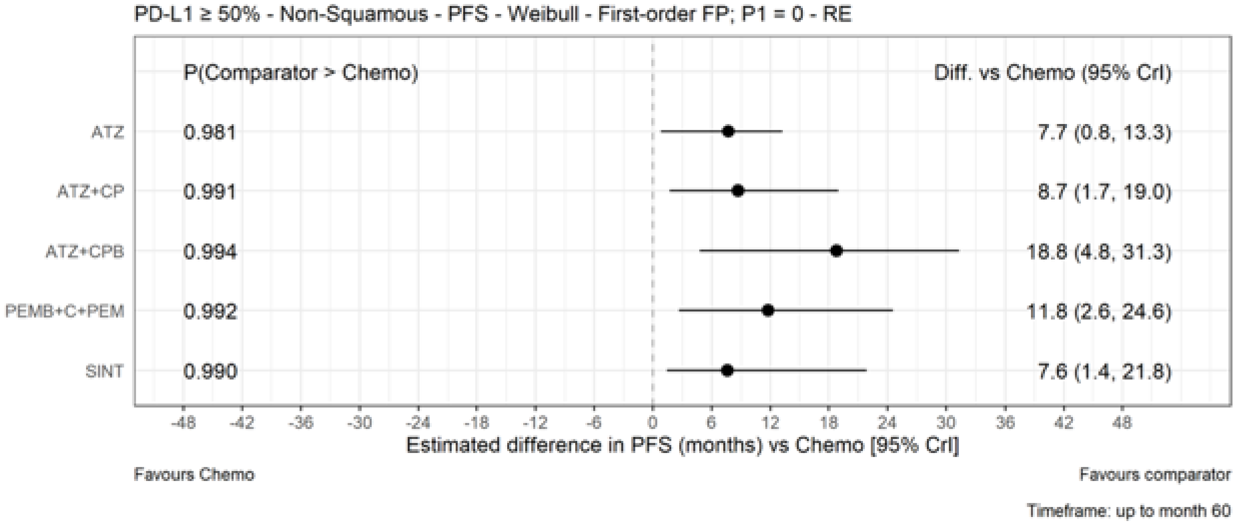


**(B)** PFS FP NMA model: HRs over time

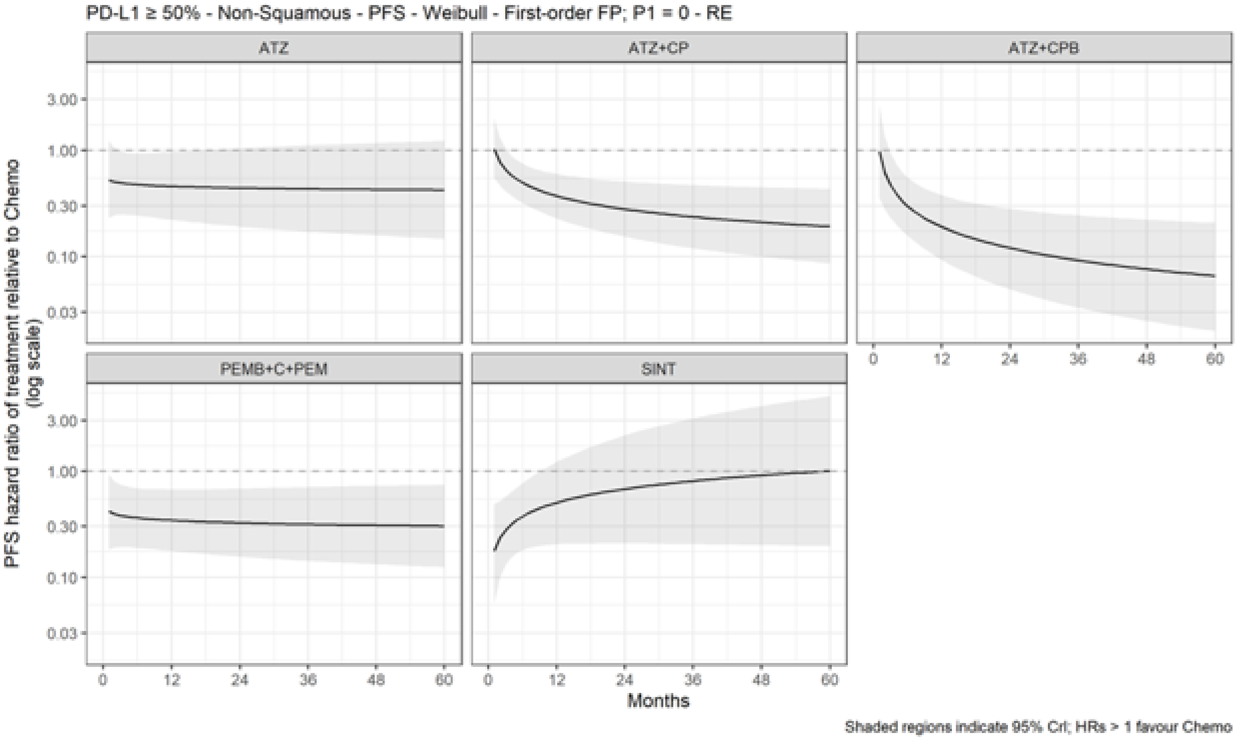

**(C)** PFS proportional hazards random effects model

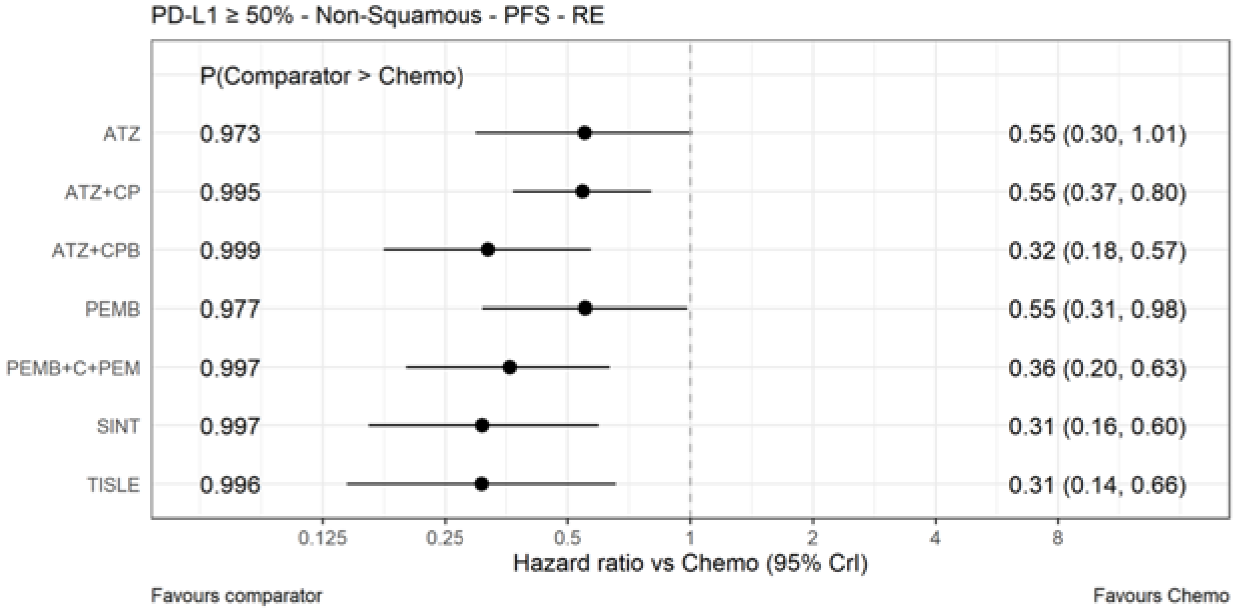


ATZ, atezolizumab; C, platinum-based chemotherapy; chemo, chemotherapy; CP, carboplatin plus paclitaxel; CPB, carboplatin plus paclitaxel plus bevacizumab; Diff., difference; PD-L1, programmed death-ligand 1; FP, fractional polynomial; NMA, network meta-analysis; PEM, pemetrexed; PEMB, pembrolizumab; PFS, progression-free survival; SINT, sintilimab; TISLE, tislelizumab.

Figure S8. ORR NMA for comparators vs chemotherapy

**(A)** Mixed-histology network

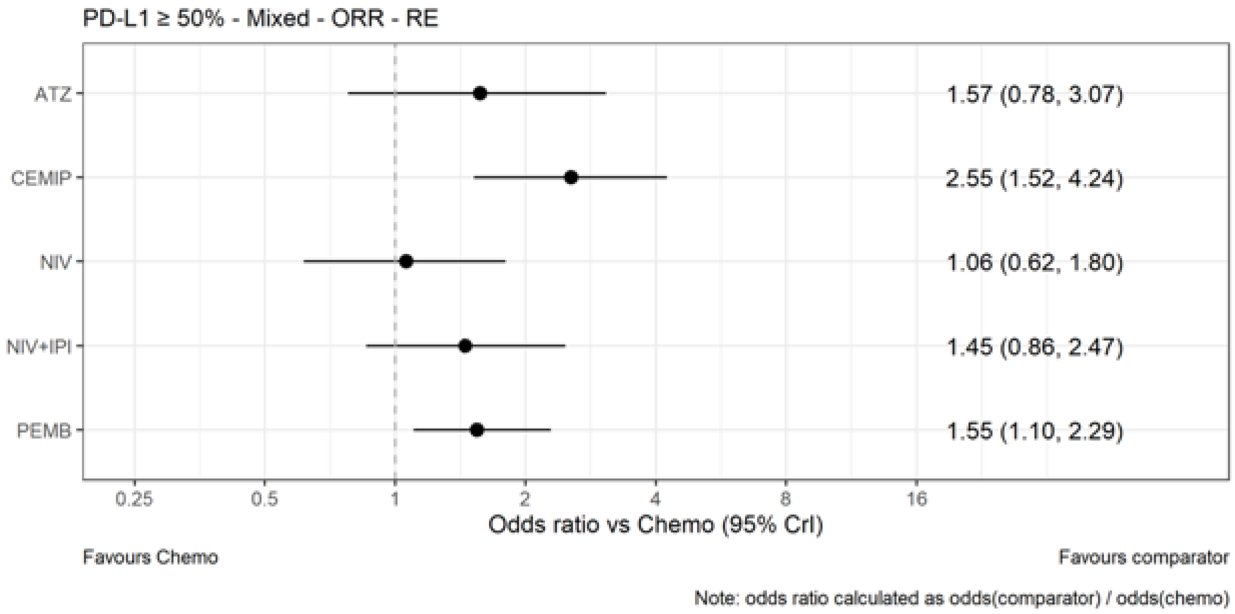


**(B)** Squamous NSCLC

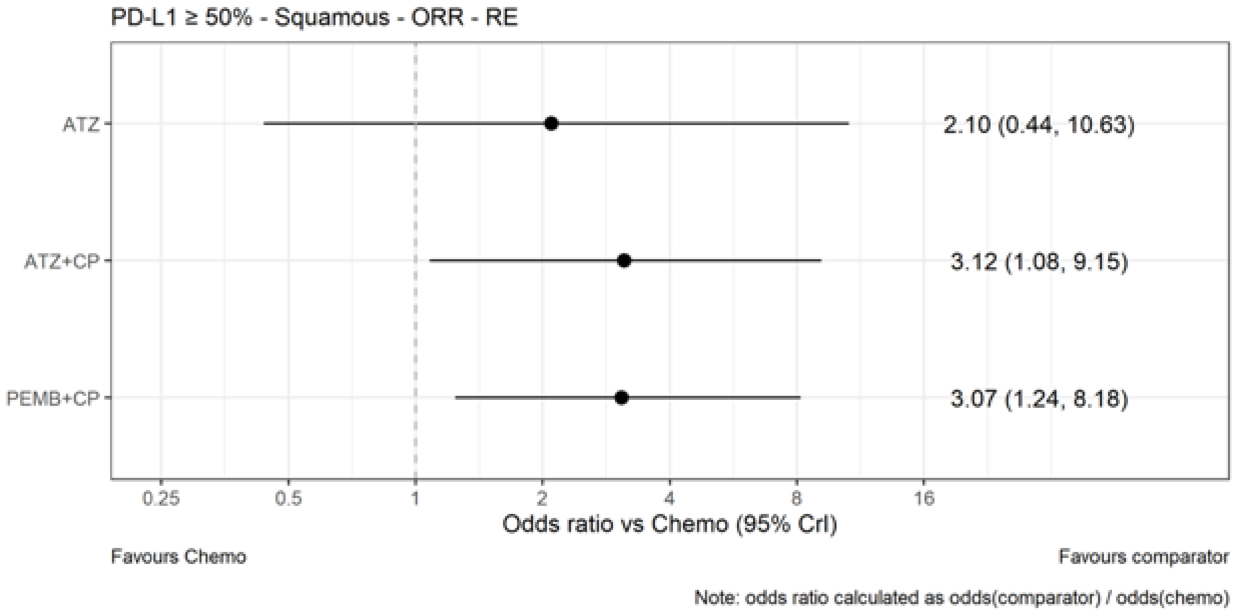

**(C)** Non-squamous NSCLC

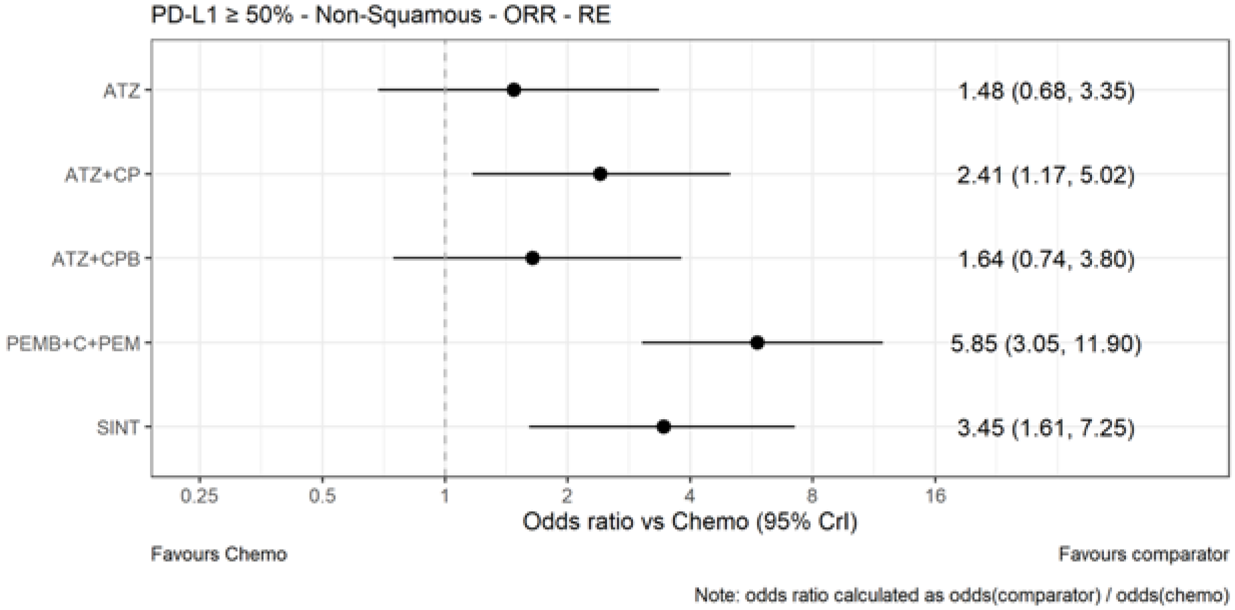


ATZ, atezolizumab; C, platinum-based chemotherapy; CEMIP, cemiplimab; CP, carboplatin plus paclitaxel; CPB, carboplatin plus paclitaxel plus bevacizumab; DURV, durvalumab; NIV, nivolumab; NIV+IPI, nivolumab plus ipilimumab; NSCLC, non-small cell lung cancer; PD-L1, programmed death-ligand 1; PEM, pemetrexed; PEMB, pembrolizumab; SINT, sintilimab.

Figure S9. Odds ratios for any treatment-related adverse event: comparators vs chemotherapy in the mixed-histology network

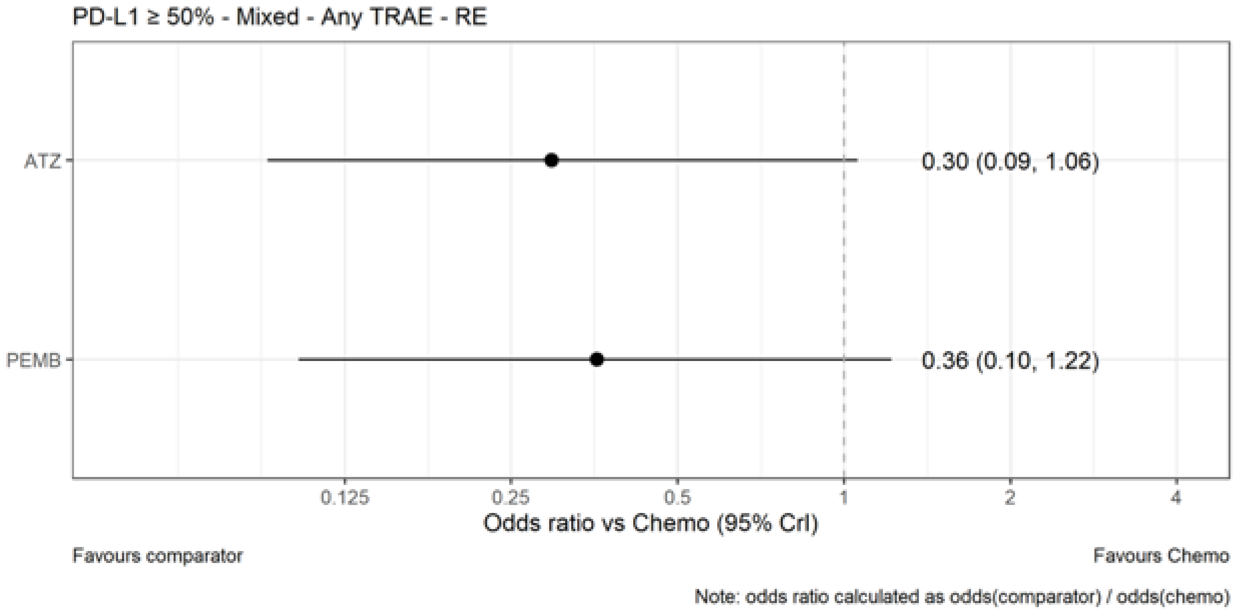


ATZ, atezolizumab; NIV, nivolumab; PD-L1, programmed death-ligand 1; PEMB, pembrolizumab.
